# Compatibility of Concurrent Aerobic and Strength Training for Skeletal Muscle Size and Function: An Updated Systematic Review and Meta-Analysis

**DOI:** 10.1007/s40279-021-01587-7

**Published:** 2021-11-10

**Authors:** Moritz Schumann, Joshua F. Feuerbacher, Marvin Sünkeler, Nils Freitag, Bent R. Rønnestad, Kenji Doma, Tommy R. Lundberg

**Affiliations:** 1 Department of Molecular and Cellular Sports Medicine, Institute of Cardiovascular Research and Sports Medicine, German Sport University, Am Sportpark Müngersdorf 6, 50933 Cologne, Germany; 2 Olympic Training Centre Berlin, Berlin, Germany; 3 Section for Health and Exercise Physiology, Department of Public Health and Sport Sciences, Inland Norway University of Applied Sciences, Elverum, Norway; 4 Sport and Exercise Science, College of Healthcare Sciences, James Cook University, Townsville, QLD Australia; 5 Division of Clinical Physiology, Department of Laboratory Medicine, Karolinska Institutet, Stockholm, Sweden; 6 Unit of Clinical Physiology, Karolinska University Hospital, Stockholm, Sweden

## Abstract

**Background:**

Both athletes and recreational exercisers often perform relatively high volumes of aerobic and strength training simultaneously. However, the compatibility of these two distinct training modes remains unclear.

**Objective:**

This systematic review assessed the compatibility of concurrent aerobic and strength training compared with strength training alone, in terms of adaptations in muscle function (maximal and explosive strength) and muscle mass. Subgroup analyses were conducted to examine the influence of training modality, training type, exercise order, training frequency, age, and training status.

**Methods:**

A systematic literature search was conducted according to the PRISMA (Preferred Reporting Items for Systematic Reviews and Meta-Analyses) guidelines. PubMed/MEDLINE, ISI Web of Science, Embase, CINAHL, SPORTDiscus, and Scopus were systematically searched (12 August 2020, updated on 15 March 2021). Eligibility criteria were as follows. Population: healthy adults of any sex and age; Intervention: supervised concurrent aerobic and strength training for at least 4 weeks; Comparison: identical strength training prescription, with no aerobic training; Outcome: maximal strength, explosive strength, and muscle hypertrophy.

**Results:**

A total of 43 studies were included. The estimated standardised mean differences (SMD) based on the random-effects model were − 0.06 (95% confidence interval [CI] − 0.20 to 0.09; *p* = 0.446), − 0.28 (95% CI − 0.48 to − 0.08; *p* = 0.007), and − 0.01 (95% CI − 0.16 to 0.18; *p* = 0.919) for maximal strength, explosive strength, and muscle hypertrophy, respectively. Attenuation of explosive strength was more pronounced when concurrent training was performed within the same session (*p* = 0.043) than when sessions were separated by at least 3 h (*p* > 0.05). No significant effects were found for the other moderators, i.e. type of aerobic training (cycling vs. running), frequency of concurrent training (> 5 vs. < 5 weekly sessions), training status (untrained vs. active), and mean age (< 40 vs. > 40 years).

**Conclusion:**

Concurrent aerobic and strength training does not compromise muscle hypertrophy and maximal strength development. However, explosive strength gains may be attenuated, especially when aerobic and strength training are performed in the same session. These results appeared to be independent of the type of aerobic training, frequency of concurrent training, training status, and age.

PROSPERO: CRD42020203777.

**Supplementary Information:**

The online version contains supplementary material available at 10.1007/s40279-021-01587-7.

## Key Points

**Table:** 

Concurrent aerobic and strength training is recommended to improve physical fitness and health; however, the compatibility of these two distinct training modes remains unclear.
In this meta-analysis, we report that concurrent training does not interfere with adaptations in maximal strength and muscle hypertrophy, regardless of the type of aerobic training (cycling vs. running), frequency of concurrent training (> 5 vs. < 5 weekly sessions), training status (untrained vs. active), mean age (< 40 vs. > 40 years), and training modality (same session vs. same day vs. different day training).
However, concurrent training may attenuate gains in explosive strength, which is exacerbated when aerobic and strength training are performed within the same training session.

## Introduction

Performing aerobic and strength training concurrently is an integrative part of physical training aimed at improving both athletic performance and health. The recommendation to perform both aerobic and strength training is important because these activities to some extent induce distinct adaptations and health benefits [1, 2]. For example, aerobic training promotes increased aerobic capacity (i.e. central adaptations) and metabolic changes in skeletal muscle, such as increased mitochondrial density and capillarisation [3]. Conversely, regular strength training results in muscle hypertrophy and increased strength and power [4] but may also improve bone mineral density [5]. The role of skeletal muscle in health maintenance has received increased attention in the last decade, with muscle tissue being understood as a secretory organ that releases several hundred myokines related to the function of other organs, such as the brain, adipose tissue, bone, liver, gut, pancreas, vascular bed, and skin [6]. In addition, the role of muscle power has recently been highlighted as being strongly associated with a lower risk of fall-related injuries in older adults [7, 8], further underlining the importance of both muscle mass and muscle function as indicators of physical health and independence in daily life.

Aside from the health perspective, many sports require the athlete to simultaneously incorporate divergent training modalities, including aerobic and strength training, into their training regimen. Considering that both athletes and recreational exercisers often perform relatively high volumes (and/or frequencies) of aerobic training alongside resistance-type training, it is pertinent to revisit the compatibility of aerobic and strength training. Aerobic training has been shown to interfere with the development of maximal strength when the overall training volume is high [9]. In contrast, no interference in maximal strength was observed when training volume was reduced to two weekly aerobic and strength training sessions, respectively [10–12]. Importantly, however, even low volumes of concurrent aerobic training have been shown to decrease gains in rapid force production [10, 13], which could translate into reduced muscle power-related benefits. Identifying additional moderators hypothesised in the literature to potentially influence neuromuscular adaptations to concurrent aerobic and strength training (such as type of aerobic training, concurrent training modality, age, and training status) could further aid in fine-tuning exercise guidelines for health and/or fitness performance.

To date, few attempts have been made to quantitatively synthesise the literature concerning concurrent aerobic and strength training. The first meta-analysis conducted a decade ago by Wilson et al. [14] showed that peak power was attenuated with concurrent training compared with strength training alone, whereas the development of muscle hypertrophy and maximal strength were not compromised. A more recent meta-analysis aimed to compare the effect of concurrent aerobic and strength training with strength training alone on the development of maximal strength in untrained, moderately trained, and trained individuals [15]. The results suggested that concurrent training may have a negative effect on lower body strength development in trained individuals but not in moderately trained or untrained individuals. While this study updated information on the effect of training status on maximal strength development, several other important outcome variables related to muscle mass and function have not been examined in a meta-analysis since 2012. Therefore, the aim of the current study was to systematically assess the compatibility of concurrent aerobic and strength training on adaptations in maximal strength, explosive strength, and muscle hypertrophy by means of pooled analyses. Subgroup analysis was also conducted to examine the influence of aerobic training type, training modality, exercise order, concurrent training frequency, age, and training status. An updated literature synthesis on this topic is relevant to physicians, physiotherapists, exercise scientists, and sports practitioners designing programmes aimed at developing both aerobic and strength qualities for health purposes, rehabilitation, and/or fitness performance.

## Methods

### Systematic Literature Search

A systematic literature search was conducted according to the PRISMA (Preferred Reporting Items for Systematic Reviews and Meta-Analyses) guidelines and was registered with PROSPERO (the International Database of Prospectively Registered Systematic Reviews in Health and Social Care; CRD42020203777). The PubMed/MEDLINE, ISI Web of Science, Embase, CINAHL, SPORTDiscus, and Scopus databases were systematically searched using a search string specifically adapted to the search requirements of each database (Table S1 in the electronic supplementary material [ESM]).

**Fig. 1:**
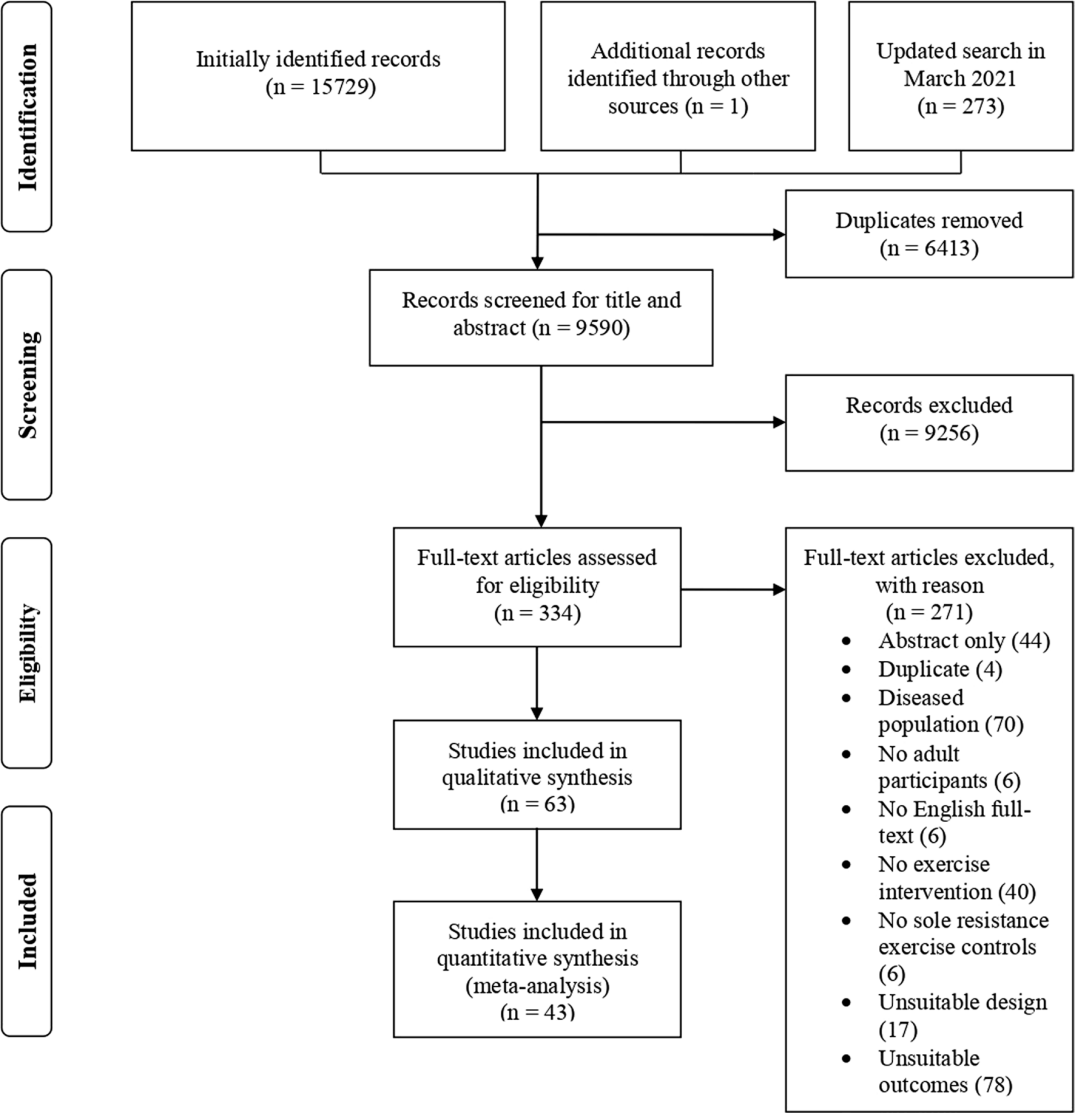
Flowchart of the search process and the study selection

The search was conducted on 12 August 2020 and updated on 15 March 2021. The literature search process was performed independently by two researchers and included saving the online search, removing duplicates, and screening titles, abstracts, and full texts. Potential conflicts were resolved by consulting with a third author. In addition, a grey literature search was performed by screening Google Scholar and the reference lists of previously identified eligible full texts. Figure 1 is a flowchart of the search process and study selection.

### Eligibility Criteria

Inclusion criteria were defined based on the PICO (Population, Intervention, Control and Outcomes) criteria [16]. The population included healthy adults with no restrictions in terms of sex and age. The intervention had to consist of supervised combined aerobic and strength training for at least 4 weeks. As a comparator, eligible studies had to include a group receiving the identical strength-training prescription with no aerobic training. Outcomes of interest included maximal strength, explosive strength, and muscle hypertrophy. The exercise tests had to be specific to the training performed. For maximal strength, both isometric and isoinertial measurements were accepted. For explosive strength, any form of jump test, isometric rate of force development (RFD), or dynamic power measurements were considered eligible. For muscle hypertrophy, objective measurements of whole-muscle cross-sectional area or muscle thickness (e.g. ultrasound, computed tomography [CT] or magnetic resonance imaging [MRI]) were required. In addition, segmental lean mass as determined by dual-energy X-ray absorptiometry (DXA) was accepted if values were reported separately for segments that were engaged in training.

Exclusion criteria included language other than English or German, abstracts and dissertations, cross-sectional studies assessing only acute exercise responses, and observational studies.

### Data Extraction

Data extraction was performed independently by two authors. The following data were extracted from each included study: (1) general characteristics (e.g. author[s], year of publication and aim of the study), (2) participant information (e.g. sample size, training status, and age), (3) intervention data for all groups (e.g. intervention duration, type of intervention), and (4) specific outcomes (e.g. measures of maximal and explosive strength and hypertrophy). If the mean and standard deviation of each group were not specified, we requested baseline and post-intervention data from the authors of the primary studies. If data were presented within a graph and no additional data were provided upon request, mean and standard deviation were extracted using WebPlotDigitizer version 4.4 (Pacifica, CA, USA) [17].

### Data Synthesis and Analyses

Standardised mean differences (SMD) were calculated, and an inverse variance-weighted random-effects model was fitted to the effect sizes (ES). Additionally, log variability ratios were calculated, and an inverse variance-weighted random-effects model was fitted to the ES. Meta-analyses were performed using R (3.6.2), RStudio (1.2.5033), and the metafor package (version 2.4.0) [18]. ES were calculated for pre-test post-test control group designs using the previously recommended raw score standardisation [19, 20]. Furthermore, the exact sampling variance of ES was computed according to recommendations [19].

Heterogeneity (i.e. *τ*^2^) was estimated using the restricted maximum-likelihood estimator [21]. To complete the heterogeneity analyses, the Q-test for heterogeneity [22] and the *I*^2^ statistic [23] were also calculated. Studentised residuals and Cook’s distances were examined to assess whether studies might be outliers and/or overly influential [24]. Studies with a studentised residual greater than the 100 × (1–0.05/(2 × *k*))th percentile of a standard normal distribution were declared potential outliers (i.e. using a Bonferroni correction with two-sided *α* = 0.05 for *k* studies included in the meta-analyses). Studies with a Cook’s distance larger than the median plus six times the interquartile range of the Cook’s distances were considered overly influential. If a study was identified as a potential outlier or overly influential, a sensitivity analysis was performed. A trim-and-fill-contour funnel plot was created to estimate the number of studies that may be missing from the meta-analysis (Fig. S1 in the ESM). We used the rank correlation test [25] and regression test [26] using the standard error of observed outcomes as predictor to check for funnel plot asymmetry.

ES from studies with more than two intervention or control groups were combined according to the Cochrane handbook recommendations [27], except for subgroup analysis when different interventions from individual studies were included in separate subgroups. If there were multiple measurements for the same outcome, only one measurement was included in the analysis, based on the following hierarchies:

- Maximal strength: (1) dynamic bilateral leg press, (2) squat, (3) unilateral isometric torque (knee extension), and (4) bilateral dynamic knee extension.
- Explosive strength: (1) jump height and (2) other measures of rapid force production as well as squat jump power and leg press power at 50% of maximal strength.
- Muscle hypertrophy: (1) whole-muscle cross-sectional area of the quadriceps femoris muscles (i.e. panoramic ultrasound, CT, MRI), (2) muscle thickness of the vastus lateralis, and (3) segmental DXA of the lower extremities.

Thus, each study was included in the final analyses with only one parameter to avoid inflating the weighting of individual studies.

Because of a lack of systematic reporting, subgroup analyses were only performed for aerobic training type (i.e. cycling vs. running), concurrent training frequency (i.e. low frequency of 4.1 ± 0.3 vs. high frequency of 6.1 ± 1.6 weekly sessions, based on 2.0 ± 0.3 vs. 3.1 ± 0.6 weekly sessions in the comparison training group), training status (i.e. untrained vs. active), mean age of the study population (18–40 vs. > 40 years), and training modality (i.e. concurrent training on different days vs. on the same day vs. in the same session). For studies comparing concurrent training in the same session, when a sufficient number of studies were available, training order was also compared (i.e. aerobic before strength exercise vs. strength before aerobic exercise). Studies were divided into subgroups based on the description in the manuscript. This was particularly true for training status, with studies classified as ‘untrained’ if participants were clearly described as ‘sedentary’, ‘previously untrained’, or ‘inactive’. Conversely, all other studies were classified as ‘active’ (i.e. ‘recreationally active’, ‘trained’, ‘well-trained’, etc.). Specific rationale for the exclusion of individual studies can be found in Table S2 in the ESM.

### Assessment of Methodological Quality

Risk of bias for the included studies was assessed independently by two reviewers using the Physiotherapy Evidence Database (PEDro) scale. The PEDro scale has previously been assessed as a valid measure of the methodological quality of randomised trials [28]. Studies scoring > 6 were classified as ‘high quality’, studies scoring 4–5 were classified as ‘medium quality’, and studies scoring < 4 were classified as ‘low quality’. The following sources of bias were considered: selection (sequence generation and allocation concealment), performance (blinding of participants/personnel), detection (blinding outcome assessors), attrition (incomplete outcome data), reporting (selective reporting), and other potential biases (e.g. recall bias). The risk-of-bias scores for the included studies are presented in Table S3 in the ESM. The mean score for scale criteria 2–11 of the PEDro scale was 4.3/10, i.e., medium quality.

## Results

### Study Characteristics

The database search identified 15,729 potentially eligible articles. After further screening and eligibility assessment, a total of 43 studies were included in the final analysis (Fig. 1). The characteristics of the studies, participants, and training interventions are summarised in Table S1 in the ESM. The meta-analysis included a total of 1090 participants, of whom 590 performed supervised combined aerobic and strength training and 500 performed strength training alone. In the included studies, cycling was the most common type of aerobic training (24 studies), followed by running (16 studies). In addition, the combination of running and cycling [9], rowing [29], and continuous repeated leg extensions [30] were each evaluated by one study.

### Maximal Strength

**Fig. 2:**
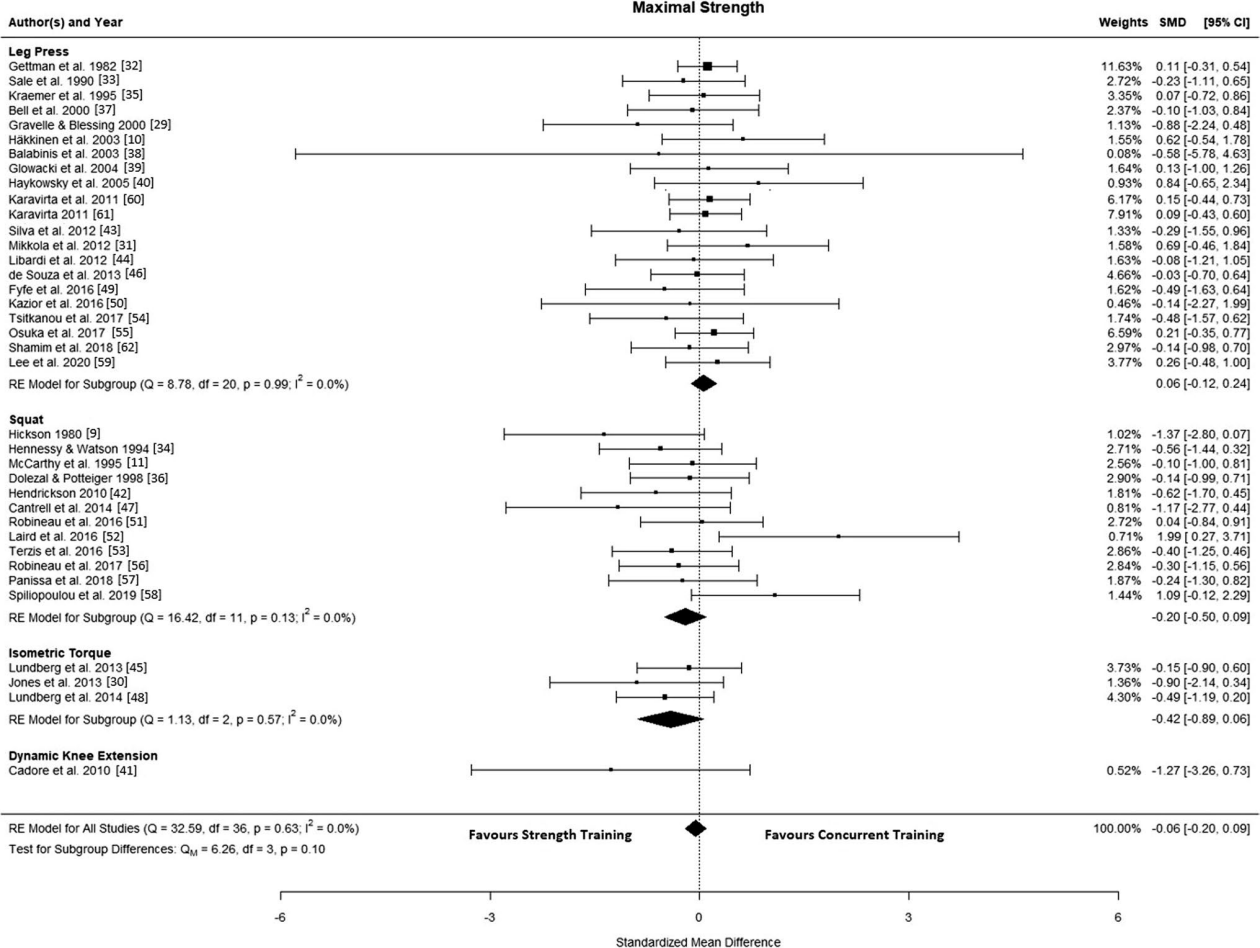
Forest plot of studies comparing differences in maximal strength. *CI* confidence interval, *RE* random effects, *SMD* standardised mean difference

The final analysis included 37 studies [9–11, 29–62], with 525 participants performing combined aerobic and strength training and 442 participants performing strength training alone. The observed SMD ranged from − 1.37 to 1.99, and the estimated average SMD based on the random-effects model was − 0.06 (95% confidence interval [CI] − 0.20 to 0.09; *p* = 0.446), indicating no interference effect of aerobic training (Fig. 2). The estimated log variability ratio based on the random-effects model was 0.05 (95% CI − 0.05 to 0.15; *p* = 0.311). According to the *Q*-test, there was no significant heterogeneity in the true outcomes (*Q*(36) = 32.591, *p* = 0.632, ${\widehat{\tau }}^{2}$= 0.000, *I*^2^ = 0.00%). An examination of the studentised residuals showed no evidence of outliers within this model, and none of the studies were overly influential.

Subgroup analyses showed no statistical differences (*p* > 0.05) (Figs. S2–S7 in the ESM).

### Explosive Strength

**Fig. 3:**
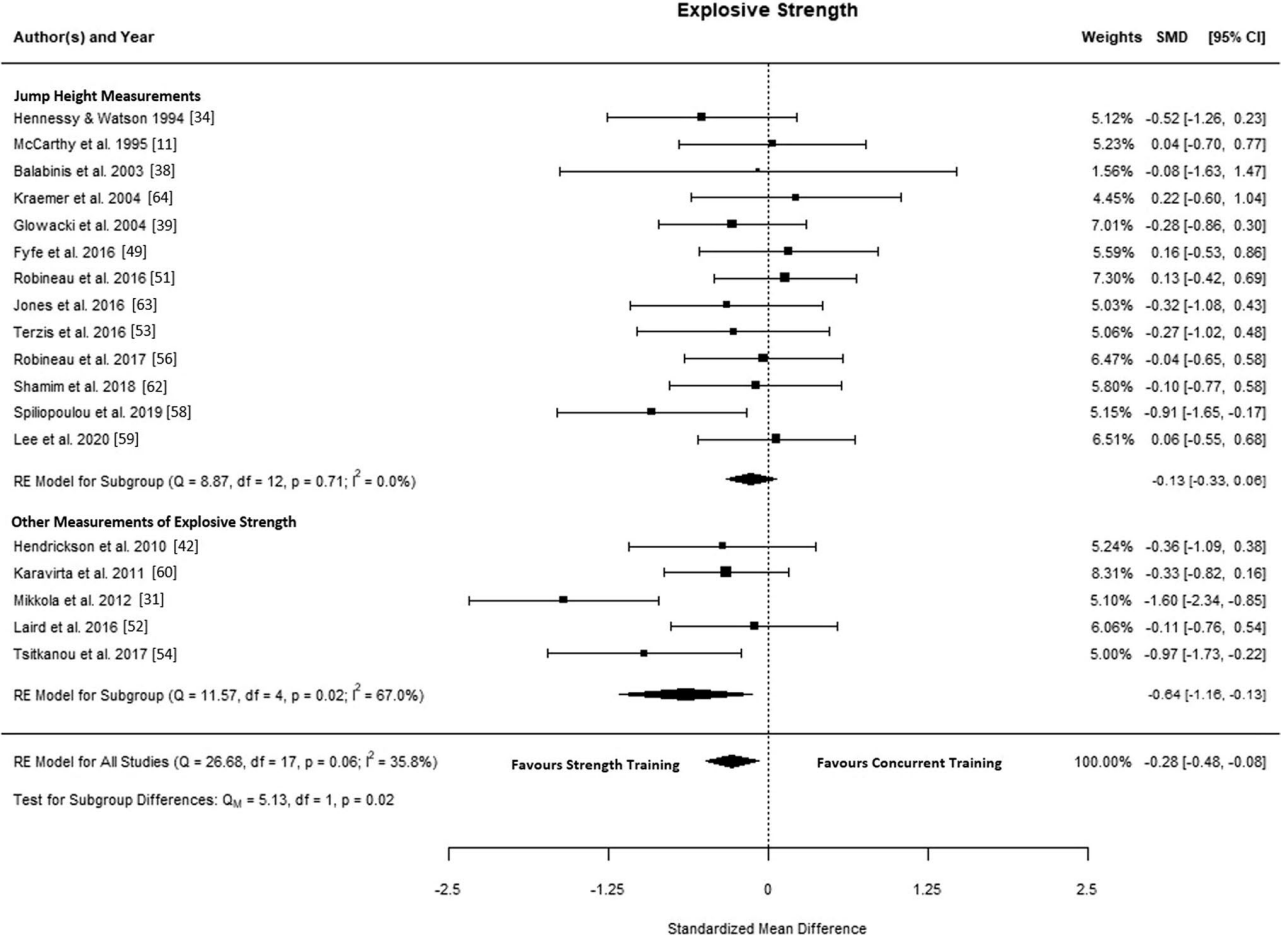
Forest plot of studies comparing differences in explosive strength. *CI* confidence interval, *RE* random effects, *SMD* standardised mean difference

The final analyses included 18 studies [11, 31, 34, 38, 39, 42, 49, 51–54, 56, 58–60, 62–64], with 270 participants performing combined aerobic and strength training and 208 performing strength training alone. The observed SMD ranged from − 1.60 to 0.22, and the estimated mean SMD based on the random-effects model was − 0.28 (95% CI − 0.48 to − 0.08; *p* = 0.007), indicating an interference effect of aerobic training (Fig. 3). The estimated log variability ratio based on the random-effects model was 0.04 (95% CI − 0.09 to 0.18; *p* = 0.533). According to the *Q* test, there was no significant heterogeneity in the true outcomes (*Q*(17) = 26.675, *p* = 0.068, ${\widehat{\tau }}^{2}$= 0.068, *I*^2^ = 35.81%). The studentised residuals highlighted Mikkola et al. [31] as a potential outlier that may have been overly influential. Sensitivity analyses revealed that excluding this study reduced the amount of observed heterogeneity to *I*^2^ = 0.00% (*Q*(16) = 13.860, *p* = 0.061, ${\widehat{\tau }}^{2}$= 0.061).

Subgroup analyses showed no statistical differences (*p* > 0.05) (Figs. S8–S11 in the ESM). When studies were grouped by type of aerobic training, the SMD was significantly in favour of strength training for cycling − 0.44 (95% CI − 0.86 to − 0.01; *p* = 0.043) but not for running (Fig. S8 in the ESM). However, after the overly influential study by Mikkola et al. [31] was removed, this effect was no longer observed (SMD − 0.27; 95% CI − 0.58 to 0.04; *p* = 0.086). A similar effect was also seen for low concurrent training frequency, with an initial SMD of − 0.45 (95% CI − 0.87 to − 0.02; *p* = 0.039) in favour of the strength training group (Fig. S9 in the ESM). After the study by Mikkola et al. [31] was removed, this reduced to − 0.25 (95% CI − 0.50 to 0.01; *p* = 0.059). Conversely, when studies were grouped by training modality, a significant interference effect was observed for studies that performed concurrent training within the same session (≤ 20 min between aerobic and strength training; SMD − 0.31; 95% CI − 0.62 to − 0.01; *p* = 0.043) but not when concurrent training was separated by at least 3 h (Fig. S11 in the ESM).

### Muscle Hypertrophy

**Fig. 4:**
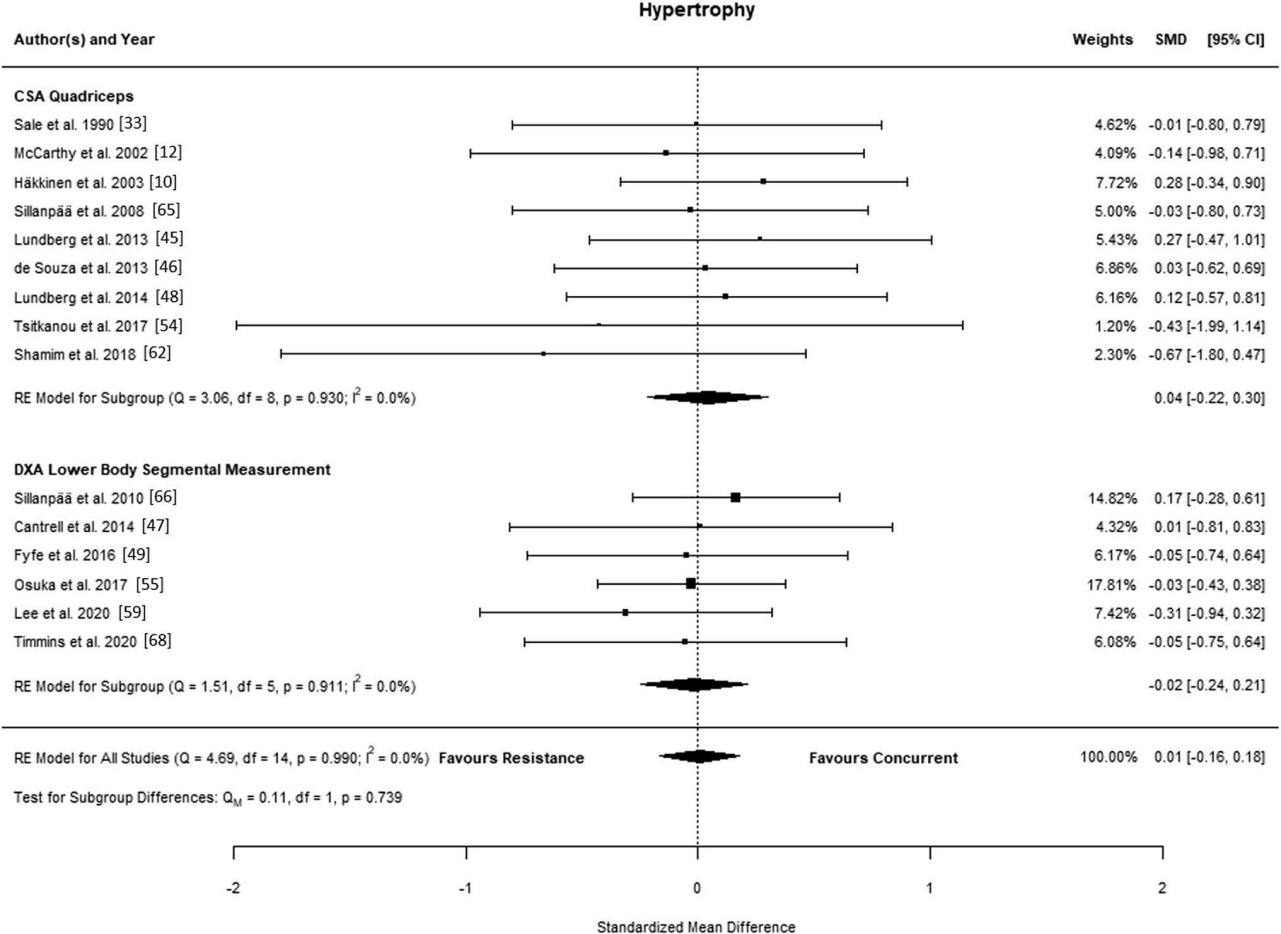
Forest plot of studies comparing differences in muscle hypertrophy. *CI* confidence interval, *CSA* cross-sectional area, *DXA* dual energy X-ray absorptiometry, *RE* random effects, *SMD* standardised mean difference

The final analyses included 15 studies [10, 11, 33, 45–47, 49, 54, 55, 59, 62, 65–68], with 201 participants performing combined aerobic and strength training and 188 performing strength training alone. The observed SMD in each trial ranged from − 0.67 to 0.28, and the estimated mean SMD based on the random-effects model was − 0.01 (95% CI − 0.16 to 0.18; *p* = 0.919), indicating no interference effect of aerobic training (Fig. 4). The estimated log variability ratio based on the random-effects model was 0.04 (95% CI − 0.11 to 0.19; *p* = 0.567). According to the *Q* test, there was no significant heterogeneity in the true outcomes (*Q*(14) = 4.687; *p* = 0.990, ${\widehat{\tau }}^{2}$= 0.000, *I*^2^ = 0.00%). An examination of the studentised residuals showed no potential outlier within this model. According to the Cook’s distances, no study could be considered overly influential. Subgroup analyses revealed no statistical differences (*p* > 0.05) (Figs. S12–S14 in the ESM).

## Discussion

The aim of this study was to provide a systematic and evidence-based appraisal of whether aerobic training interfered with adaptations to strength training in terms of muscle function (maximal and explosive strength) and whole-muscle hypertrophy. In addition, the impact of important mediating covariates such as type of aerobic training, training modality, exercise order, concurrent training frequency, age, and training status were assessed. The main finding was that concurrent aerobic and strength training did not interfere with the development of maximal strength and muscle hypertrophy compared with strength training alone. However, the development of explosive strength was negatively affected by concurrent training. Our subgroup analysis showed that this negative effect was exacerbated when concurrent training was performed within the same session, compared with when aerobic and strength training were separated by at least 3 h. No significant effects were found for other moderators, such as type of aerobic training (cycling vs. running), frequency of concurrent training (> 5 vs. < 5 weekly sessions), training status (untrained vs. active), and mean age (< 40 vs. > 40 years).

An important goal of this meta-analysis was to provide evidence that can be translated into optimised and fine-tuned exercise recommendations for fitness and health purposes. Although our results are generally consistent with those reported by Wilson et al. [14] a decade ago, these authors considered anaerobic power measures such as Wingate performance as indicators of explosive strength. Since we intentionally included only direct measures of explosive strength (i.e. jump performance, isometric RFD, and dynamic leg press power), our findings reinforce that concurrent aerobic and strength training can compromise strength qualities that require rapid neural activation.

The mechanism for compromised explosive but not maximal strength is interesting and requires further research. Our findings are supported by an early study showing that muscle hypertrophy and maximal strength were unaffected by concurrent training, whereas RFD was blunted, likely because of interference with rapid voluntary neural activation [10]. More specifically, although the maximal neural activation was not compromised, the increase in the integrated electromyographic signal during the first 500 ms was attenuated in the group performing both aerobic and strength training. Since the rate of recruitment and maximal discharge of motor neurons largely determines the maximal RFD [69], it appears that the rate of recruitment and discharge of motor units is particularly sensitive to the interference effect of aerobic training. It could be speculated that residual fatigue induced by aerobic training affects the corticospinal inputs received by the motor neurons before force is generated, which would subsequently compromise rapid force generation. The latter could potentially reduce the quality but not the quantity of strength training sessions performed concurrently with aerobic training, thereby potentially reducing the development of explosive strength but not maximal strength or muscle hypertrophy. This, in turn, could have implications for programme design, as it is apparent that concurrently improving both cardiorespiratory fitness and rapid force production through rather generic exercise recommendations presents a physiological challenge.

Consistent with this, our subgroup analysis indicated that the magnitude of interference in explosive strength development was dependent on the programming of the exercise sessions, with significant interference observed when aerobic and strength training were performed within the same training session. Previous studies have indicated that neuromuscular interference may be more pronounced when strength training is immediately preceded by aerobic training in both young [70] and older individuals [71]. However, our pooled analysis did not provide evidence for an order-specific effect but rather highlights that combining aerobic and strength training in close proximity attenuates adaptations in explosive strength regardless of exercise order. Other studies have suggested that, apart from limitations in rapid neural drive [10], adaptations in pennation angle and fascicle length [54] or patella tendon cross-sectional area [72] could be possible mechanistic explanations for these findings.

The moderators, including frequency of concurrent training, type of training, age, and training status, did not significantly influence adaptations in maximal and explosive strength, nor muscle hypertrophy. Similarly, no significant effects were observed in our analysis of log variability, indicating no within-group differences in variability after concurrent training compared with strength training alone. Our results differ from the recently published meta-analysis that focused exclusively on the effect of training status on maximal strength during concurrent training [15]. In this study, the one-repetition maximum for leg press and squat was negatively affected by concurrent training in trained individuals but not in moderately trained or untrained individuals compared with strength training alone. Moreover, their subgroup analysis suggested that the negative effect observed in trained individuals occurred only when aerobic and strength training were performed within the same training session. However, given the lack of consistent reporting, we chose not to divide the active participants into moderately or well-trained athletes, which may have diluted potential significant effects. Furthermore, albeit the exact calculations of Petré et al. [15] were not published, their analysis appears to differ from our approach. Apart from the smaller number of studies included (27 vs. 37 studies), studies consisting of multiple intervention groups with only one comparator were included multiple times in the same analysis, potentially inflating power [73]. Although the results did not reach statistical significance, our subgroup analysis for training status showed a similar direction for the SMD in trained versus untrained participants as reported by Petré et al. [15].

In other concurrent training research, numerous studies have focused on the possible interference mechanisms related to muscle hypertrophy [74]. The rationale for these studies stems from rodent and cellular models indicating possible inhibition of mechanistic target of rapamycin signalling through activation of AMP-activated protein kinase (AMPK) following aerobic exercise [75–78]. However, subsequent human studies failed to confirm these findings when examining physiological mechanisms such as metabolic stress and AMPK activation [67, 79] or protein synthesis [80] following concurrent exercise. Based on our systematic review, this is not surprising as none of the identified studies reported a significant interference effect on muscle hypertrophy. Although Wilson et al. [14] concluded from their subgroup analysis that there was a negative relationship between the ES for hypertrophy and both aerobic training frequency and duration, our results do not confirm these observations. There are several possible explanations for this inconsistency, apart from the obvious fact that our analysis was conducted almost a decade later and therefore included more studies. First, the inclusion criteria differed since Wilson et al. [14] included fibre hypertrophy as an outcome parameter and also included studies without a strength training control group. Second, we conducted our analysis based on an inverse variance-weighted random-effects model in a pre-test post-test control group design [18], whereas Wilson et al. [14] estimated the ES of each individual group, resulting in a total of 72 ES for muscle hypertrophy. The reported aerobic training duration and intensity were then correlated with ES, potentially leading to significant positive correlations.

Although the current meta-analysis provides updated and novel information, some limitations should be acknowledged. First, it should be noted that the majority of the included studies were only classified as of medium quality (mean PEDro score 4.3 ± 0.9), and seven studies were of low quality. However, it is important to note that it may not be possible to achieve all items related to blinding in exercise trials. In addition, poor reporting quality may have biased the outcome of this ranking. Thus, more importance can possibly be given to the studentised residuals and the Cook’s distance [24]. Furthermore, meta-analyses are generally limited to the information provided within the included individual studies. Even though we contacted authors to request additional information, the response rate was low. Therefore, to avoid speculation, we decided to include only clearly defined moderators. For example, aerobic exercise intensity was not included because the included studies did not provide consistent information. However, it is possible that aerobic exercise intensity may impact on the compatibility of aerobic and strength training. A meta-analysis examining the effects of concurrent high-intensity interval training (HIIT) and strength training reported that lower body strength development was compromised by concurrent training compared with strength training alone, even though the authors noted that a possible negative effect on lower body strength may be ameliorated by the inclusion of running-based HIIT and longer intermodal rest periods [81]. This was further supported by a recent narrative review reporting that HIIT could minimise the risk of neuromuscular interference and that this effect was even more pronounced when HIIT was replaced with sprint-interval training [82]. However, it should be acknowledged that previous research appears to indicate that the overall health benefits of concurrent training, apart from muscle function and size, appear to be greater than those obtained with isolated training of either aerobic or strength training [83, 84] and that the overall risk of interference effects is rather low. Therefore, most individuals, including recreational athletes, can enjoy complementary benefits from incorporating both aerobic and strength training into their training programme.

## Conclusion

This updated meta-analysis shows that concurrent aerobic and strength training does not interfere with the development of maximal strength and muscle hypertrophy compared with strength training alone. This appears to be independent of the type of aerobic training (cycling vs. running), frequency of concurrent training (> 5 vs. < 5 weekly sessions), training status (untrained vs. active), and mean age (< 40 vs. > 40 years). However, the evidence of reduced development of explosive strength with concurrent training, particularly when aerobic and strength training are performed in the same session, suggests that practitioners who prioritize explosive strength may benefit from separating aerobic and strength training to achieve optimal adaptations.

## Supplementary Information

Below is the link to the electronic supplementary material.

Supplementary file1 (PDF 2017 KB)

## Declarations

### Code availability

The code will be available upon reasonable request.
