# Supplementary material for: Compatibility of Concurrent Aerobic and Strength Training for Skeletal Muscle Size and Function: An Updated Systematic Review and Meta-Analysis
Source: Sports Med. 2021 Nov 10;52(3):601–12. doi: 10.1007/s40279-021-01587-7 (PMC8891239; doi:10.1007/s40279-021-01587-7)
Supplement: Supplementary file 1 — Supplementary file1 (PDF 2017 KB) [file 40279_2021_1587_MOESM1_ESM.pdf]

Online Supplemental Data

Table S1. Databases and the corresponding search string.

Database	Search string
PubMed/MEDLINE	(("Humans"[MeSH Terms] AND (("concurrent"[All Fields] OR "combin*"[All Fields]) OR "simultaneous"[All Fields])) AND ("strength"[All Fields] OR "resistance"[All Fields])) AND ("endurance"[All Fields] OR "aerobic"[All Fields])
ISI Web of Science	TS=Humans OR people AND concurrent training OR combined training AND strength exercise OR resistance exercise OR resistance training OR strength training AND aerobic training OR aerobic exercise OR endurance training OR endurance exercise
Embase	TS=Humans OR people AND concurrent training OR combined training AND strength exercise OR resistance exercise OR resistance training OR strength training AND aerobic training OR aerobic exercise OR endurance training OR endurance exercise
CINAHL	TS=Humans OR people AND concurrent training OR combined training AND strength exercise OR resistance exercise OR resistance training OR strength training AND aerobic training OR aerobic exercise OR endurance training OR endurance exercise
SPORTDiscus	TS=Humans OR people AND concurrent training OR combined training AND strength exercise OR resistance exercise OR resistance training OR strength training AND aerobic training OR aerobic exercise OR endurance training OR endurance exercise
Scopus	TS=Humans OR people AND concurrent training OR combined training AND strength exercise OR resistance exercise OR resistance training OR strength training AND aerobic training OR aerobic exercise OR endurance training OR endurance exercise

Table S2. Excluded/included studies where results from the same study groups were published more than once.

Included study	Excluded study	Outcome
Hendrickson et al. (2010) [1]	Nindl et al. (2010) [2]<br>DuPont et al. (2017) [3]	Maximal strength
Libardi et al. (2012) [4]	Libardi et al. (2011) [5]	Maximal strength
Bell et al. (2000) [6]	Horne et al. (1997) [7]<br>Putman et al. (2004) [8]	Maximal strength
Karavirta et al. (2011a) [9]& Karavirta (2011b) [10]*	Holviala et al. (2010 & 2012)[11, 12] , Ahtiainen et al. (2009) [13], Sillanpää et al. (2009a, 2009b, & 2012) [14–16], Karavirta et al. (2009, 2013) [17, 18]	Maximal strength
Karavirta et al. (2011a) [9]	Holviala et al. (2012) [12]	Explosive strength
Sillanpää et al. (2008) [19]	Ahtiainen et al. (2009) [13]	Hypertrophy
Sillanpää et al. (2010) [20]	Sillanpää et al. (2009b) [14]	Hypertrophy

*The dissertation of Karavirta was used for data extraction since upon request we were informed that maximal strength values are reported in their entirety. NB: The studies of Chtara et al. (2008) [21], Coleman et al. (1996) [22], Dudley et al. (1985) [23], Gergley et al. (2009) [24], Lambert et al. (2015) [25], Leveritt et al. (2003) [26] and Peres Campos et al. (2013) [27] were found eligible for data extraction. However, the presented data were not suitable for further analysis (i.e. data were presented in percentage change, no post-test data available, outcome was measured, but not reported). The same applied to the study of Häkkinen et al. (2003) [28], where the data on changes in rate of force development were only presented as percentage changes. Similarly, Jones et al. (2016) [29] reported maximal strength data only as percentage changes.

Table S3. PEDro Scores for the included studies.

**Authors (year)**	**PEDro Scale criterion number**										**Total**
	1	2	3	4	5	6	7	8	9	10	11	
Balabinis et al. (2003) [30]	No	0	0	0	0	0	0	1	0	1	1	**3**
Bell et al. (2000) [6]	No	1	0	1	0	0	0	0	0	1	1	**4**
Cadore et al. (2010) [31]	Yes	1	0	1	0	0	0	1	0	1	1	**5**
Cantrell et al. (2014) [32]	Yes	1	0	1	0	0	0	1	0	1	1	**5**
de Souza et al. (2013) [33]	Yes	1	0	1	0	0	0	1	0	1	1	**5**
Dolezal & Potteiger (1998) [34]	No	1	0	1	0	0	0	0	0	1	1	**4**
Fyfe et al. (2016) [35]	No	1	0	1	0	0	0	0	0	1	1	**4**
Gettman et al. (1982) [36]	No	1	0	1	0	0	0	0	0	1	1	**4**

Glowacki et al. (2004) [37]	Yes	1	0	0	0	0	0	1	0	1	1	**4**
Gravelle & Blessing (2000) [38]	Yes	1	0	0	0	0	0	1	0	1	1	**4**
Häkkinen et al. (2003) [28]	No	0	0	1	0	0	0	0	0	1	1	**3**
Haykowski et al. (2005) [39]	No	1	0	0	0	0	0	0	0	1	1	**3**
Hendrickson et al. (2010) [1]	Yes	1	0	1	0	0	0	1	0	1	1	**5**
Hennessy & Watson (1994) [40]	No	1	0	1	0	0	0	0	0	1	1	**4**
Hickson (1980) [41]	No	0	0	0	0	0	0	0	0	1	1	**2**
Jones et al. (2013) [42]	No	1	0	1	0	0	0	0	0	1	1	**4**
Jones et al. (2016) [29]	No	1	0	1	0	0	0	1	0	1	1	**5**
Karavirta et al. (2011) [9]	Yes	1	0	1	0	0	0	1	0	1	1	**5**
Karavirta (2011) [10]	Yes	1	0	1	0	0	0	1	0	1	1	**5**
Kazior et al. (2016) [43]	No	0	0	1	0	0	0	0	0	1	1	**3**
Kraemer et al. (1995) [44]	No	1	1	1	0	0	0	1	0	1	1	**6**
Kraemer et al. (2004) [45]	No	1	1	1	0	0	0	0	0	1	1	**5**
Laird et al. (2016) [46]	Yes	0	0	1	0	0	0	1	0	1	1	**4**
Lee et al. (2020) [47]	No	0	0	1	0	0	0	1	0	1	1	**4**
Libardi et al. (2012) [4]	No	1	0	1	0	0	0	1	0	1	1	**5**
Lundberg et al. (2013) [48]	No	1	0	1	0	0	0	1	0	1	1	**5**

Lundberg et al. (2014) [49]	No	1	0	1	0	0	0	1	0	1	1	**5**
McCarthy et al. (1995) [50]	No	1	0	1	0	0	0	0	0	1	1	**4**
McCarthy et al. (2002) [51]	No	1	0	1	0	0	0	1	0	1	1	**5**
Mikkola et al. (2012) [52]	No	0	0	1	0	0	0	1	0	1	1	**4**
Osuka et al. (2017) [53]	Yes	1	1	1	0	0	0	1	1	1	1	**7**
Panissa et al. (2018) [54]	No	1	0	1	0	0	0	1	0	1	1	**5**
Robineau et al. (2016) [55]	No	1	0	1	0	0	0	0	0	1	1	**4**
Robineau et al. (2017) [56]	No	1	0	1	0	0	0	0	0	1	1	**4**
Sale et al. (1990) [57]	No	1	0	1	0	0	0	0	0	1	1	**4**
Shamim et al. (2018) [58]	No	0	0	1	0	0	0	0	0	1	1	**3**
Sillanpää et al. (2008) [19]	Yes	1	0	1	0	0	0	1	0	1	1	**5**
Sillanpää et al. (2010) [20]	Yes	1	0	1	0	0	0	0	0	1	1	**4**
Silva et al. (2012) [59]	No	1	0	1	0	0	0	1	0	1	1	**5**
Spiliopoulou et al. (2019) [60]	No	0	0	1	0	0	0	1	0	1	1	**4**
Terzis et al. (2016) [61]	No	1	0	1	0	0	0	1	0	1	1	**5**
Timmins et al. (2020) [62]	No	0	0	1	0	0	0	0	0	1	1	**3**
Tsitkanou et al. (2017) [63]	No	0	0	1	0	0	0	1	0	1	1	**4**

1 = eligibility criteria were specified, 2 = subjects were randomly allocated to groups, 3 = allocation was concealed, 4 = groups were similar at baseline, 5 = all subjects were blinded, 6 = therapist who administered therapy/training were blinded, 7 = all assessors who measured key outcomes were blinded, 8 = measurement of key outcomes were obtained from more than 85% of the subjects, 9 = subjects for whom outcome measures were available received the treatment or control condition as allocated or, otherwise for at least one key outcome was analysed by "intention to treat", 10 = results of between-group statistical comparisons were reported for at least on key outcome, 11 = study provides both point measures and measures of variability, score: 0 = no, 1 = yes.

Table S4. Study characteristics of included studies.

Author(s)	Participants	Training modality		Strength Training	Aerobic Training	Outcome
Balabinis et al. (2003) [30]	ST = 7, CT = 7; college basketball athletes	7 weeks,<br>ST: 4 sessions/wk<br>CT: 8 sessions/wk (4 × ST + 4 × AT)	Same day CT, AT was performed 7 hours before ST	Exercise: half squat, bench press, leg press, latissimus-pull;<br>Set configuration: weeks 1-3, 3-6 × 3-6 repetitions at 75-95% 1RM; week 4-5, 4-5 × 5-8 repetitions at 70% 1RM; plyometric (2 × 15 repetitions of multiple cone hops, tuck jumps, push up depth jump, handstand depth jump), weeks 6-7, 3 × 30-40 repetitions at 40% 1RM	Running: week 1, 5 miles at 70% HRmax; weeks 2-7 multiple sets of interval runs, strides and full-speed runs (30-500 m) at intensities > 85% HRmax	1RM Leg Press ↑[a b]<br>Vertical Jump Height ↑[a b]
Bell et al. (2000) [6]	ST = 11, CT = 13; physically active university students	12 weeks,<br>ST: 3 sessions/wk<br>CT: 6 sessions/wk (3 × ST + 3 × AT)	Different day CT, ST and AT performed on alternating days	Exercise: single leg - leg press, leg flexion/extension, calf raises, bench press, latissimus-pull, shoulder press, biceps curls;<br>Set configuration: intensity increased by 4% every 3 weeks, mean of 2-6 × 4-12 repetitions at 72-84% 1RM	Cycling: continuous cycling at 30 min per session and progressed to 42 min per session (4 min increase every 4 weeks), interval sessions were performed once a week, 4-6 × 3 min at 90% $\dot{V}O_2$max, and 3 min of active recovery between each bout (increased by 1 set every 4 weeks)	1RM Leg Press ↑[a b]
Cadore et al. (2010) [31]	ST = 8, CT = 8; healthy elderly men	12 weeks,<br>ST: 3 sessions/wk<br>CT: 6 sessions/wk (3 × ST + 3 × AT)	Same session CT, AT was performed before ST	Exercise: inclined leg-press, knee extension, leg curl, bench press, latissimus pull down, seated row, triceps curl, biceps curl and abdominal exercises;<br>Set configuration: weeks 1-4, 2 × 18-20 RM; weeks 5-7, 2 × 12-14 RM; weeks 8-10, 3 × 12-14 RM; weeks 11-12, 3 × 6-8 RM	Cycling: weeks 1-2, 20 min at 80% HR at ventilatory threshold, consistently progressing to 30 min at 95% in week 7, and 6 × 4 min at 100% in weeks 11-12	1RM Knee extension ↑[a b]
Cantrell et al. (2014) [32]	ST = 7, CT = 7; recreationally active, college students	12 weeks,<br>ST: 2 sessions/wk<br>CT: 4 sessions/wk (2 × ST + 2 × AT)	Different day CT, ST and AT performed on alternating days	Exercise: squat, bench press, leg extension, leg curl, pull down, shoulder press;<br>Set configuration: 3 × 4-6 repetitions at 85% 1RM	Cycling: weeks 1-4, 4-6 × of a modified 20 sec Wingate protocol; starting with 4 sets progressing by 1 set every 4 weeks until 6 sets	1RM Squat ↑[a b]<br>DXA Lower Body ↑[a b]

de Souza et al. (2013) [33]	ST = 11, CT = 11; active physical education students	8 weeks,<br>ST: 2 sessions/wk<br>CT: 4 sessions/wk (2 × ST + 2 × AT)	Same session CT, ST and AT performed on alternating days	Exercise: knee extension, knee flexion, leg-press 45°, knee extension and knee flexion;<br>Set configuration: 3-5 × 6-12 RM	Running: intensity was 80-100% of the maximal velocity at V̊O2max; 15-20 × of 60 sec b1outs with 45-90 sec rest	1RM Leg Press ↑[a b]<br>CSA QF ↑[a b]
Dolezal & Potteiger (1998) [34]	ST= 10, CT= 10, physically active	10 weeks,<br>ST: 3 sessions/wk<br>CT: 6 sessions/wk (3 × ST + 3 × AT)	Same session CT, ST before AT	Exercise: Olympic free weights and universal machines;<br>Set configuration: weeks 1-2, 3 × 10-15 RM; weeks 3-10, 1 set 10-12 RM, 1 set 8-10 RM, 1 set 4-8 RM	Running: weeks 1-2, 25 min at 65% of HRmax; weeks 3-6, 35 min at 65-75% HRmax; weeks 7-10, 40 min at 75-85% HRmax	1RM Squat ↑[a b]
Fyfe et al. (2016) [35]	ST = 9, HIT+ST = 9, MICT+ST = 10; recreationally active	8 weeks,<br>ST: 3 sessions/wk<br>CT: 6 sessions/wk (3 × ST + 3 × AT)	Same session CT, AT performed 10 min before ST	Exercise: one session: leg press, bench press, seated row, leg extension and leg curl exercises were included; other session: leg press, flat dumbbell press, latissimus pulldown, dumbbell lunges and leg curl exercises;<br>Set configuration: 3-5 × 4-14 repetitions at 65-90% 1RM	Cycling: HIT: 2 min intervals at intensities between 120 and 150% of the lactate threshold (LT) and 1 min recovery; MICT: 15 - 33 min at a relative intensity between 80 and 100% of the LT	1RM Leg Press ↑[a b]<br>DXA Lower Body ↑[a]<br>CMJ Height →
Gettman et al. (1982) [36]	ST = 30, CT = 34; N/A	12 weeks,<br>ST: 3 sessions/wk<br>CT: 6 sessions/wk (3 × ST + 3 × AT)	Same session CT, combination of 30 sec stationary training + 30 sec running after each station, total workout time was 30 min	Exercise: squat, shoulder press, knee flexion, bench press, leg press, elbow flexion, hyperextension, elbow extension, sit ups, vertical fly;<br>Set configuration: 12 - 15 repetitions at 40% 1RM within 30 seconds duration at each of the stations, rest period was 15sec, total workout time 22.5min	Running: 30 seconds at >60 % HRmax, performed after 30 seconds of ST	1RM Leg Press ↑ [a b]
Glowacki et al. (2004) [37]	ST = 13, CT = 16; untrained	12 weeks,<br>ST: 2-3 sessions/wk<br>CT: 5 sessions/wk (2-3 × ST + 2-3 × AT)	Combination of same and different day CT	Exercise: leg press, leg curl, standing calf raises, bench press, latissimus pull-down, dumbbell military press, barbell curl, abdominal crunch;<br>Set configuration: 3 × 6-10 repetitions at 50-85% 1RM	Running: 20-40 min on an indoor treadmill or outdoor track at 65-80% of HRR; intensity and/or duration were increased biweekly	1RM Leg Press ↑[a b]<br>Vertical Jump Height →

Gravelle & Blessing (2000) [38]	ST = 6, CT1 S+E = 6, CT2 E+S = 7; active	11 weeks, ST: 3 sessions/wk CT: 6 sessions/wk (3 × ST + 3 × AT)	Same session CT, CT1 performed ST before AT, CT2 performed AT prior to ST	Exercise: leg press, squat, knee extension/flexion, straight leg deadlifts, heel raise; Set configuration: weeks 1-2, 2 × 10 RM; weeks 3-4, 3 × 10 RM; weeks 5-9, 4 × 10 RM; weeks 10-11, 4 × 6-8 RM	Rowing: 70% $\dot{V}O_2max$; subjects began with 25 min per session in week 1; exercise duration was increased by 5 min per week until 45 min were achieved; in addition, stroke rate was increased by 1 stroke per minute per week after half of the training period was completed	1RM Leg Press ↑[a b]
Häkkinen et al. (2003) [28]	ST = 16, CT = 11; healthy	21 weeks, ST: 2 sessions/wk CT: 4 sessions/wk (2 × ST + 2 × AT)	Different day CT, AT and ST performed on separate days	Exercise: leg press, bilateral/unilateral knee extension, bench press, triceps push, latissimus pull, sit-up, trunk extensor, elbow flexion, leg abduction/adduction; Set configuration: week 1-7, 3-4 × 10-15 repetitions at 50-70% 1RM, weeks 8-14, 3-5 × 5-6 repetitions at 60-80% 1RM and 3-5 × 8-12 repetitions at 50-60% 1RM for leg extensor exercises and 3-5 × 10-12 RM for all other exercises, weeks 15-21 subjects performed either higher loads with 4-6 × 3-6 repetitions at 70-80% 1RM and loads with 4-6 × 8-12 repetitions at 50-60% 1RM for leg extensor exercises and 3-5 × 8-12 RM for all other exercises	Cycling or walking; weeks 1-7, 30 min of continuous cycling or walking below aerobic threshold level; weeks 8-14, 45 min, including 15 min below the aerobic threshold, 10 min between aerobic-anaerobic thresholds, 5 min above the anaerobic threshold and 15 min again under the aerobic threshold or 60 min continuous work below aerobic threshold; weeks 15 - 21, 60 min of exercise including 15 min under the aerobic threshold, 2 × 10 min between aerobic-anaerobic thresholds, 2 × 5 min above anaerobic threshold and the final 15 min under aerobic threshold or 60-90 min continuous work below aerobic threshold	1RM Leg Press ↑[a b] MRI QF ↑[a b]
Haykowski et al. (2005) [39]	ST = 7, CT = 6; N/A	12 weeks, ST: 3 sessions/wk CT: 6 sessions/wk (3 × ST + 3 × AT)	N/A	Exercise: leg press, leg extension, leg curl, chest press shoulder press, latissimus pull-down, triceps push down, unilateral arm curl; Set configuration: week 1, 2 × 10 repetitions at 50% 1RM, increased by 2.5% every week up to 75% 1RM	Cycling: continuous cycling at 60-80% HRR; week 1, 15 min, progressing by 2.5 min every week up to 42.5 min	1RM Leg Press ↑[a b]

Hendrickson et al. (2010) [1]	ST = 18, CT = 15; recreationally active	8 weeks,<br>ST: 3 sessions/wk<br>CT: 6 sessions/wk (3 × ST + 3 × AT)	Same session CT, "light" ST-days always corresponded to AT-days	Exercise: various free weight/machine exercises, targeting the major upper- and lower body muscles;<br>Set configuration: "light" (3 × 12 repetitions), "moderate" (3 × 8-10 repetitions) or "heavy" (3 × 3-8 repetitions) with daily and weekly load variation in non-linear order	Running: continuous running for 20-30 min at 70-85% HRmax or sprint-type interval consisting of 400, 800, 1200 and 1600 m runs near maximal intensity with a 1:1 load-recovery-ratio	1RM Squat ↑[a b]<br>Squat Jump Peak Power →
Hennessy & Watson (1994) [40]	ST = 9, CT = 10; active	8 weeks,<br>ST: 3 sessions/wk<br>CT: 7 sessions/wk (3 × ST + 4 × AT)	Combination of same and different day CT	Lower body exercise: bench press and back squat exercise;<br>Set configuration: 2 × 10 repetitions at 65% 1RM, load was progressively increased by 5% every week until 6 sets with maximal number of repetition possible for 105% 1RM were performed by the end of the intervention; upper body exercise hamstring curls, latissimus pulldown, shoulder press, arm curls and abdominal crunches and lunge, upright row, dumbbell flies, triceps press, calf raise and bent knee sit ups;<br>Set configuration: 3 × 10 repetitions	Running: 2 days, continuous low intensity runs at 70% HRmax with gradually increased duration from 20 min to 60 min over 8 weeks;<br>3 day, "fartlek" running with 5 min jog + fast 200 m strides / 200 m easy jog repeats, followed by a series of fast short distance strides between 30-100 m, each followed by recovery jog, finishing with 5 min jogging; fartlek duration was increased from 15 min in week 1 to 35 min in week 8; 4 day continuous running at 85% of HRmax, duration increased from 20 min in the first week to 40 min	1RM Half-Squat ↑[a b c]<br>Vertical Jump Height ↑[a]
Hickson (1980) [41]	ST = 8, CT = 7; recreationally active	10 weeks<br>ST: 3 sessions/wk<br>CT: 11 sessions/wk (5 × ST + 6 × AT)	Combination of same and different day CT	Exercise: squat and knee flexion/extension exercises;<br>Set configuration: 3-5 × 5 repetitions, and 3 × 5 repetitions leg press, 3 × 20 repetitions of calf raise, all exercises were performed with at least at 80% of 1RM, in addition deadlift and sit-ups were performed	Cycling and running: 3 of 5 days, interval training on ergometer with 6 × 5 min cycling at a work rate that approached subjects $\dot{V}O_2$max, separated by 2 min rest, other days, continuous running for 30-40 min as fast as possible,	1RM Half-Squat ↑[N/A]

Jones et al. (2013) [42]	ST = 6, CT1 = 6, CT2 = 6; recreationally active	6 weeks,<br>ST: 3 sessions/wk<br>CT1: 4 sessions/wk (3 × ST + 1 × AT)<br>CT2: 6 sessions/wk (3 × ST + 3 × AT)	Combination of same session and different day CT, CT1 performed 3 ST sessions per week with every third session followed by AT;<br>CT2 performed 3 St sessions per week with each immediately followed by AT	Exercise: unilateral leg extensions; Set configuration: 5 × 6 repetitions at 80 ± 5% of their individual isometric MVC with 3 minutes rest intervals	Repeated leg extensions: 30 min of repeated isokinetic unilateral leg extensions at 30 ± 5% of individual MVC for that session; frequency: 1 second per muscle action	MVC Leg Extension Torque ↑ (for ST and CT2 only) [a b d]
Jones et al. (2016) [29]	ST = 8, CT1 = 8, CT2 = 8; recreationally active	6 weeks,<br>ST: 3 sessions/wk<br>CT1: 4 sessions/wk (3 × ST + 1 × AT)<br>CT2: 6 sessions/wk (3 × ST + 3 × AT)	combination of same session and different day concurrent training, CT1 performed 3 ST sessions per week with every third session followed by an AT session;<br>CT2 performed 3 ST sessions per week followed by AT	Exercise: various free weight/machine exercises, targeting the major upper- and lower body muscles; training sessions were designated as "compound", "pull" or "push"); Set configuration: week 1-3, 4 × 8 repetitions at 80% 1RM; weeks 3-6, 5 × 6 repetitions at 85% 1RM	Running: continuous treadmill running at 70% of peak running velocity at $\dot{V}O_2max$	CMJ Height ↑[a b c]

Karavirta et al. 2011 [9]	ST = 25, CT = 25; untrained	21 weeks, ST: 2 sessions/wk CT: 4 sessions/wk (2 × ST + 2 × AT)	Different day CT	Exercise: leg press, knee extension, leg curl, seated calf raises, hip abduction/adduction, bench press, biceps curl, triceps push-down, latissimus pull-down, abdominal crunch, seated back extension; each training session included 2 exercises for the leg extensor muscles and 5 exercises for the other main muscle groups; Set configuration week 1-7, 3 × 12-20 repetitions at 40-60% 1RM; weeks 8-14 subjects performed 2-4 × 5-12 repetitions at 60-80% 1RM; weeks 15-21, 2-4 × 5-8 repetitions at 70-85% of 1RM; 20% of leg press, knee extension and bench press exercises were performed with 40-50% of 1RM with 5-8 repetitions as rapidly as possible	Cycling: weeks 1-7, continuous cycle ergometer for 30 min below aerobic threshold; in addition, during weeks 5-7, subjects did three sessions,10 min interval with intensity above aerobic threshold; weeks 8-14, either 45 min of exercise including 10 min of work between the aerobic-anaerobic thresholds and 5 min above anaerobic threshold, in addition to 15 min warm up and 15 min cool down below aerobic threshold or 60 min continuous work below aerobic threshold; weeks 15-21, 60 min of exercise including 2 × 10 min intervals between the aerobic-anaerobic thresholds, 2 × 5 min of work above anaerobic threshold and 30 min below the aerobic threshold or 90 min continuous work below aerobic threshold	1RM Leg Press ↑[a b] 50%1RM Leg Press Power ↑[a b]
Karavirta 2011 [10]	ST = 25, CT = 23; untrained	21 weeks, ST: 2 sessions/wk CT: 4 sessions/wk (2 × ST + 2 × AT)	Different day CT	Exercise: leg press, knee extension, leg curl, seated calf raises, hip abduction/adduction, bench press, biceps curl, triceps push-down, latissimus pull-down, abdominal crunch, seated back extension; each training session included 2 exercises for the leg extensor muscles and 5 exercises for the other main muscle groups; Set configuration: week 1-7, 3 × 12-20 repetitions at 40-60% 1RM; weeks 8-14 subjects performed 2-4 × 5-12 repetitions at 60-80% 1RM; weeks 15-21, 2-4 × 5-8 repetitions at 70-85% of 1RM; 20% of leg press, knee extension and bench press exercises were performed with 40-50% of 1RM with 5-8 repetitions as rapidly as possible	Cycling: weeks 1-7, continuous bicycle ergometer for 30 min below aerobic threshold; in addition, during weeks 5-7, subjects did three sessions, 10 min interval with intensity above aerobic threshold; weeks 8-14, either 45 min of exercise including 10 min of work between the aerobic-anaerobic thresholds and 5 min above anaerobic threshold, in addition to 15 min warm up and 15 min cool down below aerobic threshold or 60 min continuous work below aerobic threshold; weeks 15-21, 60 min of exercise including 2 × 10 min intervals between the aerobic-anaerobic thresholds, 2 × 5 min of work above anaerobic threshold and 30 min below the aerobic threshold or 90 min continuous work below aerobic threshold	1RM Leg Press →

Kazior et al. (2016) [43]	ST = 7, CT = 9; healthy	7 weeks,<br>ST: 2-4 sessions/wk<br>CT: 4-8 sessions/wk (2-4 × ST + 2-4 × AT)	same session CT, AT was performed before ST training	Exercise: leg press;<br>Set configuration: 70% of 1RM and this load was increased by 5-7% every 3rd or 4th training session, the number of sets was increased from 4 at week 1 to 6 at week 5 and number of repetitions in each set decreased from 12 to 8 with a 3-min rest between sets. The subjects were guided to perform each repetition at a set pace, i.e., with concentric and eccentric phases of 2 seconds each	Cycling: ergometer cycling at 63 ± 1.2% of $\dot{V}O_2max$ with training intensity being increased progressively every two weeks; interval cycling at 95 ± 1.8% of $\dot{V}O_2max$ in the final three weeks	1RM Leg Press ↑[a b]
Kraemer et al. (1995) [44]	ST = 9, CT1 = 9, CT2 = 9; healthy	12 weeks,<br>ST: 4 sessions/wk<br>CT: 8 sessions/wk (4 × ST + 4 × AT)	same day CT, AT was performed before ST after 5 to 6 hours rest	Exercise: various free weight/machine exercises, targeting the major upper- and lower body muscles);<br>Set configuration: 2-3 × 10-25 RM and 3-5 × 5-10 RM	Running: long-distance or sprint-interval workouts; long distance training, running as far as possible in 40 min; sprint-interval training, interval distances ranging from 200-800 m and intensities between 95-100% of $\dot{V}O_2max$; exercise-to-rest ratio progressed from 1:4 to 1:0.5	1RM Leg Press ↑[a b c]
Kraemer et al. (2004) [45]	ST = 9, CT1 = 9, CT2 = 9; healthy	12 weeks,<br>ST: 4 sessions/wk<br>CT: 8 sessions/wk (4 × ST + 4 × AT)	Same day CT, AT was performed before ST after 5 to 6 hours rest	Exercise: various free weight/machine exercises, targeting the major upper- and lower body muscles;<br>Set configuration: 2-3 × 10-25 RM and 3-5 × 5-10 RM	Running: long-distance or sprint-interval workouts; long distance training, running as far as possible in 40 min; sprint-interval training, interval distances ranging from 200-800 m and intensities between 95-100% of $\dot{V}O_2max$; exercise-to-rest ratio progressed from 1:4 to 1:0.5	CMJ Height ↑[a b]
Laird et al. (2016) [46]	ST = 14, CT = 12; recreationally active	11 weeks,<br>ST: 3 sessions/wk<br>CT: 6 sessions/wk (3 × ST + 3 × AT)	Same day CT, ST was performed before AT after 4 hours	Exercise: back squats, bent over row, bench press, sit ups, squat jumps, deadlift, standing press and back extension exercises;<br>Set configuration: load variation occurred within an undulating periodization model with loads ranging between 4-5 × 3-5 repetitions and 3 × 10 repetitions at 70-87.5% of 1RM	Running: 8 work intervals of treadmill running for 20 seconds with 10 second passive recovery for 4 min. 2 sessions were completed at 110, 115, and 120% of $v\dot{V}O_2max$, once all 8 intervals were completed for 2 consecutive sessions, treadmill velocity was increased by 3%	1RM Squat ↑[a b]<br>RFD Squat →

Lee et al. (2020) [47]	ST = 9, CT1 = 10, CT2 = 10; moderately active	9 weeks, ST: 3 sessions/wk CT: 6 sessions/wk (3 × ST + 3 × AT)	Same day CT, AT before ST (CT1), ST before AT (CT2), separated by 3 hours	Exercise: leg press, bench press, seated row, leg extension, leg curl, dumbbell chest press, lat. pulldown, lunges; Set configuration: intensity progressed from 3 to 4 sets and from 12 to 6 RM	Cycling: intervals separated by 1 min recovery periods; volume and intensity were progressed by increasing the number of intervals from 8 to 13 and intensity from 40% to 90% of the difference between power at LT and peak power	1RM Leg Press ↑[a b] CMJ Height →[c] DXA Lower Body ↑[a b]
Libardi et al. (2012) [4]	ST = 11, CT = 11; inactive	16 weeks, ST: 3 sessions/wk CT: 6 sessions/wk (3 × ST + 3 × AT)	Same session CT, ST was performed before AT	Exercise: leg press, leg extension, and leg curl, bench press, lateral pulldown, lateral raise, triceps pushdown, arm curl, and basic abdominal crunch; Set configuration: week 1-8, 3 × 10 RM, week 9-16, 3 × 8 RM	Running: 30 min of walking or running in an athletic track, 10 min under VT intensity, 20 min at VT intensity, 20 min above VT and under respiratory compensation point intensity, 10 min under VT intensity; after 8 weeks, training session duration was maintained und subjects performed 5 min under VT intensity, 10 min above VT, 10 min at respiratory compensation point intensity, 5 min under VT intensity	1RM Leg Press ↑[a b]
Lundberg et al. (2013) [48]	same person different limbs n = 10; moderately trained	5 weeks, ST: 2-3 sessions/wk CT: 5-6 sessions/wk (2-3 × ST + 3 × AT)	Same day CT, AT was performed 6 hours before ST	Exercise: knee extensions for both limbs; Set configuration: 4 × 7 RM	Cycling: 40 min continuous one-legged cycle ergometer exercise at 70% of peak power; after 40 min workload increased by ~20 W until failure	Knee extension ↑[a b] MRI QF Volume ↑[a b]
Lundberg et al. (2014) [49]	same person different limbs n = 10; moderately trained	5 weeks, ST: 2-3 sessions/wk CT: 5-6 sessions/wk (2-3 × ST + 3 × AT)	Same session CT, AT was performed before ST with 15 min recovery in between	Exercise: knee extensions for both limbs; Set configuration: 4 × 7 RM	Cycling: 40 min continuous one-legged cycle ergometer exercise at 70% of peak power; after 40 min workload increased by ~20 W until failure	Knee extension → MRI QF Volume ↑[b]
McCarthy et al. (1995) [50]	ST = 10, CT = 10; sedentary healthy	10 weeks, ST: 3 sessions/wk CT: 6 sessions/wk (3 × ST + 3 × AT)	Same session CT, AT and ST performed in alternating order	Exercise: squats, bench press, standing curls, knee extension, leg curl, lat. Pull-down, overhead press, heel raise; Set configuration: 3 x 6 RM	Cycling: 50 min continuous cycling ergometer exercise at 70% HRR	1RM Squat ↑[a b] Vertical Jump Height ↑[a b]

McCarthy et al. (2002) [51]	ST = 10, CT = 10; sedentary healthy	10 weeks,<br>ST: 3 sessions/wk<br>CT: 6 sessions/wk (3 × ST + 3 × AT)	Same session CT, AT and ST performed in alternating order	Exercise: squats, bench press, standing curls, knee extension, leg curl, lat. Pull-down, overhead press, heel raise;<br>Set configuration: 3 x 6 RM	Cycling: 50 min continuous cycling ergometer exercise at 70% HRR	CSA Thigh Extensor ↑[a b]
Mikkola et al. (2012) [52]	ST = 16, CT = 11; healthy	21 weeks,<br>ST: 2 sessions/wk<br>CT: 4 sessions/wk (2 × ST + 2 × AT)	Different day CT	Exercise: leg press, knee extension, leg curl, seated calf raises, hip abduction/adduction, bench press, biceps curl, triceps push-down, latissimus pull-down, abdominal crunch, seated back extension;<br>Set configuration: week 1-7, 3-4 × 10-15 repetitions at 50-70% 1RM, weeks 8-14, 2-5 × 5-12 repetitions at 50-80% 1RM, weeks 15-21, 2-5 × 3-12 repetitions at 50-85% of 1RM, additionally 20% of leg press and knee extension exercises were performed with 50-60% of 1RM as rapidly as possible	Cycling: weeks 1-7, continuous bicycle ergometer for 30 min below aerobic threshold; in addition, during weeks 5-7, subjects did three sessions,10 min interval with intensity above aerobic threshold; weeks 8-14, either 45 min of exercise including 10 min of work between the aerobic-anaerobic thresholds and 5 min above anaerobic threshold, in addition to 15 min warm up and 15 min cool down below aerobic threshold or 60 min continuous work below aerobic threshold; weeks 15-21, 60 min of exercise including 2 × 10 min intervals between the aerobic-anaerobic thresholds, 2 × 5min of work above anaerobic threshold and 30 min below the aerobic threshold or 90 min continuous work below aerobic threshold	1RM Leg Press ↑[a b]<br>RFD Knee Extension ↑[a c]
Osuka et al. (2017) [53]	ST = 28, CT = 28; healthy	12 week,<br>ST: 2 sessions/wk<br>CT: 4 sessions/wk (2 × ST + 2 × AT)	Same session CT, AT was performed before ST	Exercise: chest press, leg extension, leg curl, and leg press exercises;<br>Set configuration: weeks 1-4, 3 × 10 repetitions at 30-50% 1RM, weeks 5-8, to 3 × 12 repetitions at 50-70% 1RM, weeks 9-12, 3 × 12 repetitions at >70% 1RM	Cycling: continuous cycle ergometer at 40-50% of $\dot{V}O_2$peak; training volume was gradually increased from 20 min in weeks 1-4 to 25 min in weeks 5-8, up to 30 min in weeks 9-12	1RM Leg Press ↑[a b]
Panissa et al. (2018) [54]	ST = 11, CT = 8; physically active	8 weeks,<br>ST: 2 sessions/wk<br>CT: 4 sessions/wk (2 × ST + 2 × AT)	Same session CT, AT was performed before ST	Exercise: bench press, half-squat, triceps extension, leg extension, seated row, leg curl, and arm curl exercises;<br>Set configuration: 3 × 8-12 repetitions	Running: high-intensity interval protocol; treadmill running for one minute at 100% maximal aerobic speed with one minute of passive recovery until they completed 5 km	1RM Half Squat ↑[a b]

Robineau et al. (2016) [55]	ST = 10, CT1 = 15, CT2 = 11, CT3 = 12; amateur rugby players	7 weeks, ST: 2 sessions/wk CT: 4 sessions/wk (2 × ST + 2 × AT)	Same session CT, (CT1), same day CT, 6 hours between the sessions (CT2), different day CT, separated by 24 hours (CT3), ST was always performed before AT	Exercise lower limbs: half-squat and leg press, set configuration: 3-4 × 3-10 repetitions, upper limbs, bench press and bench row; Set configuration: weeks 1-2, 3-4 × 10 repetitions at 70% of 1RM; weeks 3-7, 3-4 × 3-6 repetitions at 80-90% 1RM; core training, plyometric and eccentric hamstring exercises were performed as warm up and complementary exercises	Running: 3 × 6 min repetitions of high intensity (15 seconds/15 seconds) interval training on a field; 15 seconds at 120% of their individual MAV, 15 seconds of passive recovery; a 5 min warm-up, distance was increased when HR was lower than 90% HRmax	1RM Half Squat [N/A]<br>CMJ Height [N/A]
Robineau et al. (2017) [56]	ST = 11, CT1 = 10, CT2 = 9; amateur rugby players	8 weeks, ST: 2 sessions/wk CT: 4 sessions/wk (2 × ST + 2 × AT)	Different day CT	Exercise: half-squat and leg press, bench press and bench row; Set configuration: weeks 1-2, 3-4 × 10 repetitions at 70% of 1RM; weeks 3-7, 3-4 × 3-6 repetitions at 80-90% 1RM; core training, plyometric and eccentric hamstring exercises were performed as warm up and complementary exercises	Running: short intervals, for CT1 group, 2 × of 30 seconds runs at 100% of MAV, separated by 30 seconds of active recovery at 50% MAV; distance was increased if HR was lower than 90% HRmax; for CT2 group sprint intervals, including repetitions of 30 seconds all-out-running with 4 min of passive recovery, each session was preceded by 15 min warm up	1RM Half Squat [N/A]<br>CMJ Height [N/A]
Sale et al. (1990) [57]	n = 8; same person different limb	22 weeks. ST: 3 sessions/wk CT: 6 sessions/wk (3 × ST + 3 × AT)	Same session CT, AT was performed before ST training	Exercise: unilateral leg press; Set configuration: 6 × 15-20 repetitions	Cycling: 5 × of 3 min one-legged cycling on ergometer at 90-100% $\dot{V}O_2$max; training was performed with the assigned leg for AT (group A) or alternately with both legs (group B) with 1-3 min rest in between	1RM Leg Press ↑[a b]<br>CSA QF ↑[a b]
Shamim et al. (2018) [58]	ST = 10, CT = 12; recreationally active	8 weeks, ST: 3 sessions/wk CT: 6 sessions/wk (3 × ST + 3 × AT)	Different day CT	Exercise: leg press, knee extension and bench press; Set configuration: 60-98% of 1RM, 3 min rest, progressive overload was applied by periodically manipulating the number of sets, repetitions, and relative intensity of load throughout the 12-week program	Cycling: hill-simulation ride of varying intensity (25-110% of MAP), moderate-intensity continuous training at 50% MAP, moderate-intensity interval training at 70% MAP and high-intensity interval training at 100% MAP. Moderate-intensity intervals were separated by a 60 second recovery period at ~ 40% MAP, 2.5:1 or 5:1 work-to-rest ratio, separated by 20- to 60 seconds recovery periods at ~ 40% MAP	1RM Leg Press ↑[a b]<br>US VL Thickness ↑[a b]<br>CMJ Height →

Sillanpää et al. (2008) [19]	ST = 13, CT = 15; healthy	21 weeks,<br>ST: 2 sessions/wk<br>CT: 4 sessions/wk (2 × ST + 2 × AT)	Different day CT	Exercise: leg press, knee extension, leg curl, seated calf raises, hip abduction/adduction, bench press, biceps curl, triceps push-down, latissimus pull-down, abdominal crunch, seated back extension;<br>Set configuration: week 1-7, 3 × 12-20 repetitions at 40-60% 1RM, weeks 8-14, 2-4 × 5-12 repetitions at 60-80% 1RM, weeks 15-21, 2-4 × 5-8 repetitions at 70-85% of 1RM, additionally 20% of leg press, knee extension and bench press exercises were performed with 40-50% of 1RM and 5-8 repetitions as rapidly as possible	Cycling: weeks 1-7, continuous bicycle ergometer for 30 min below aerobic threshold; weeks 8-14, either 45 min of exercise including 15 min of work above aerobic threshold or 60 min continuous cycling or Nordic walking below aerobic threshold; weeks 15-21, 60 min of exercise including 2 × 10 min intervals between the aerobic-anaerobic thresholds, 2 × 5 min of work above anaerobic threshold and 30 min below the aerobic threshold or 90 min continuous cycling or Nordic walking below aerobic threshold	US VL Thickness ↑(in the first 10 weeks) [a b]
Sillanpää et al. (2010) [20]	ST = 22, CT = 22; N/A	21 weeks,<br>ST: 2 sessions/wk<br>CT: 4 sessions/wk (2 × ST + 2 × AT)	Different day CT	Exercise: leg press, knee extension, leg curl, seated calf raises, hip abduction/adduction, bench press, biceps curl, triceps push-down, latissimus pull-down, abdominal crunch, seated back extension;<br>Set configuration: week 1-7, 3 × 12-20 repetitions at 40-60% 1RM, weeks 8-14, 2-4 × 5-12 repetitions at 60-80% 1RM, weeks 15-21, 2-4 × 5-8 repetitions at 70-85% of 1RM, additionally 20% of leg press, knee extension and bench press exercises were performed with 40-50% of 1RM and 5-8 repetitions as rapidly as possible	Cycling: weeks 1-7, continuous bicycle ergometer for 30 min below aerobic threshold; weeks 8-14, either 45 min of exercise including 15 min of work above aerobic threshold or 60 min continuous cycling or Nordic walking below aerobic threshold; weeks 15-21, 60 min of exercise including 2 × 10 min intervals between the aerobic-anaerobic thresholds, 2 × 5 min of work above anaerobic threshold and 30 min below the aerobic threshold or 90 min continuous cycling or Nordic walking below aerobic threshold	DXA Lower Body ↑[a b]

Silva et al. (2012) [59]	ST = 12<br>CT1 continuous running =10,<br>CT2 interval running = 11,<br>CT3 continuous cycling = 11; physically active	11 weeks,<br>ST: 2 sessions/wk<br>CT: 4 sessions/wk (2 × ST + 2 × AT)	Same session CT, AT was performed before ST	Exercise: inclined leg press, knee extension, leg curl, bench press, inverted fly, upright row, and sit-ups;<br>Set configuration: 2-3 × 8-18 RM; recovery time between sets was 120 seconds	Running or cycling: aerobic exercise for 20-30 min; continuous training at 95 % HRmax of the VT (± 3 bpm); interval running: 1 min bouts at $v\dot{V}O_2max$, with 1 min of active recovery at 50 % of $v\dot{V}O_2max$; subjects in all groups performed the same duration of aerobic exercise	1RM Leg Press ↑[a b]
Spiliopoulou et al. (2019) [60]	ST = 10, CT = 10; physical education students	6 weeks<br>3 sessions per week in the ST group<br>6 sessions per week in the CT group (3 × per week ST + 3 × per week AT)	Same session CT, ST was performed before AT	Exercise: half squat, CMJ and drop jumps<br>Set configuration: 2 of 3 days per week consisted of 6 × 2 fast eccentric-only half squats, with each set followed by 3 CMJs; training load for half-squat exercise was gradually increased from 40% 1RM in weeks 1-2, to 55% 1RM in weeks 3-4, up to 65% 1RM in weeks 5-6; training on the other day included 8 × 3 CMJs and 8 × 3 Drop Jumps (DJ); initial height for DJs was gradually increased from 20 cm in weeks 1-2, to 30 cm in weeks 3-4, to 40 cm in weeks 5-6	Cycling: high intensity intervals on stationary bicycle, 10 bouts of 1 min cycling at MAP (mean: 141 ± 13 W) with 1 min passive rest; workload increased 5% every week	1RM Half Squat ↑[a b]<br>CMJ Height ↑[a c]
Terzis et al. (2016) [61]	ST = 10, CT = 10; physical education students	6 weeks,<br>ST: 3 sessions/wk<br>CT: 6 sessions/wk (3 × ST + 3 × AT)	Same session CT, ST was performed before AT	Exercise: half squat, CMJ and drop jumps<br>Set configuration: 2 of 3 days per week consisted of 6 × 2 fast eccentric-only half squats, with each set followed by 3 CMJs; training load for half-squat exercise was gradually increased from 40% 1RM in weeks 1-2, to 55% 1RM in weeks 3-4, up to 65% 1RM in weeks 5-6; training on the other day included 8 × 3 CMJs and 8 × 3 Drop Jumps (DJ); initial height for DJs was gradually increased from	Running: walking/jogging for 30 min at 60–70 % of maximum heart rate; training intensity was gradually increased from initially 1.8 m × s^{-1} to 2.2 m × s^{-1}	1RM Half Squat ↑[a b]<br>CMJ Height ↑[a c]

				20 cm in weeks 1-2, to 30 cm in weeks 3-4, to 40 cm in weeks 5-6		
Timmins et al. (2020) [62]	ST = 10, CT = 12; recreationally active	12 weeks, ST: 3 sessions/wk CT: 6 sessions/wk (3 × ST + 3 × AT)	Different day CT	Exercise: leg press, deadlift, knee extension, barbell lunge, hip thruster, and stiff-legged-deadlift exercises; Set configuration: 2-5 × 2-15 repetitions at 70-97.5% 1RM	Cycling: hill simulation rides of varying intensity (25–110% of MAP), moderate-intensity continuous training at 50% MAP, moderate-intensity interval training at 70% MAP and high-intensity interval training at 100% MAP. Moderate-intensity intervals were separated by a 60 second recovery period at ~ 40% MAP, 2.5:1 or 5:1 work-to-rest ratio, 20- to 60-s recovery periods, completed at ~ 40% MAP	DXA Lower Body ↑[a b]
Tsitkanou et al. (2017) [63]	ST = 11, CT = 10; university students	8 weeks, ST: 2 sessions/wk CT: 4 sessions/wk (2 × ST + 2 × AT)	Same session CT, ST was performed before AT	Exercise: inclined leg press and half squat exercises; Set configuration: 4 × 6 repetitions at 80% of 6 repetitions in the first week, load was increased by 2.0-2.5% in every training; in addition 2 × 10 repetitions of abdominal crunches, lateral crunches and dorsal raises were performed during warm up prior to training	Cycling: 10 × of 60 seconds at 100% of maximal aerobic power at 55-60 rpm; training load increased by +2%	1RM Leg Press ↑[a b] US QF Volume ↑[a b] RFD Leg Press (→ in ST, ↓ in CT)[c]

ST = strength training, AT = aerobic training, CT = concurrent training, RM = repetition maximum, DXA = dual energy X-ray absorptiometry, CSA = cross-sectional area, QF = quadriceps femoris, MRI = magnetic resonance imaging, US = ultrasound, VL = vastus lateralis, CMJ = countermovement jump, RFD = rate of force-development, HR = heart rate, HRmax = maximal heart rate, HRR =heart rate reserve, VT = ventilatory threshold, MVC = maximal voluntary contraction, $\dot{V}O_2max$ = maximal oxygen consumption, $v\dot{V}O_2max$ = velocity at $\dot{V}O_2max$, MAV = maximal aerobic velocity, MAP = maximal aerobic power, N/A = not available, [a] significant pre vs. post difference in the strength training group, [b]significant pre vs. post difference in the concurrent training group, [c]between-group difference in favour of strength training, [d]between-group difference in favour of strength training and concurrent training (group 2) compared to concurrent training (group 1), significant difference is set at ($p \leq 0.05$).

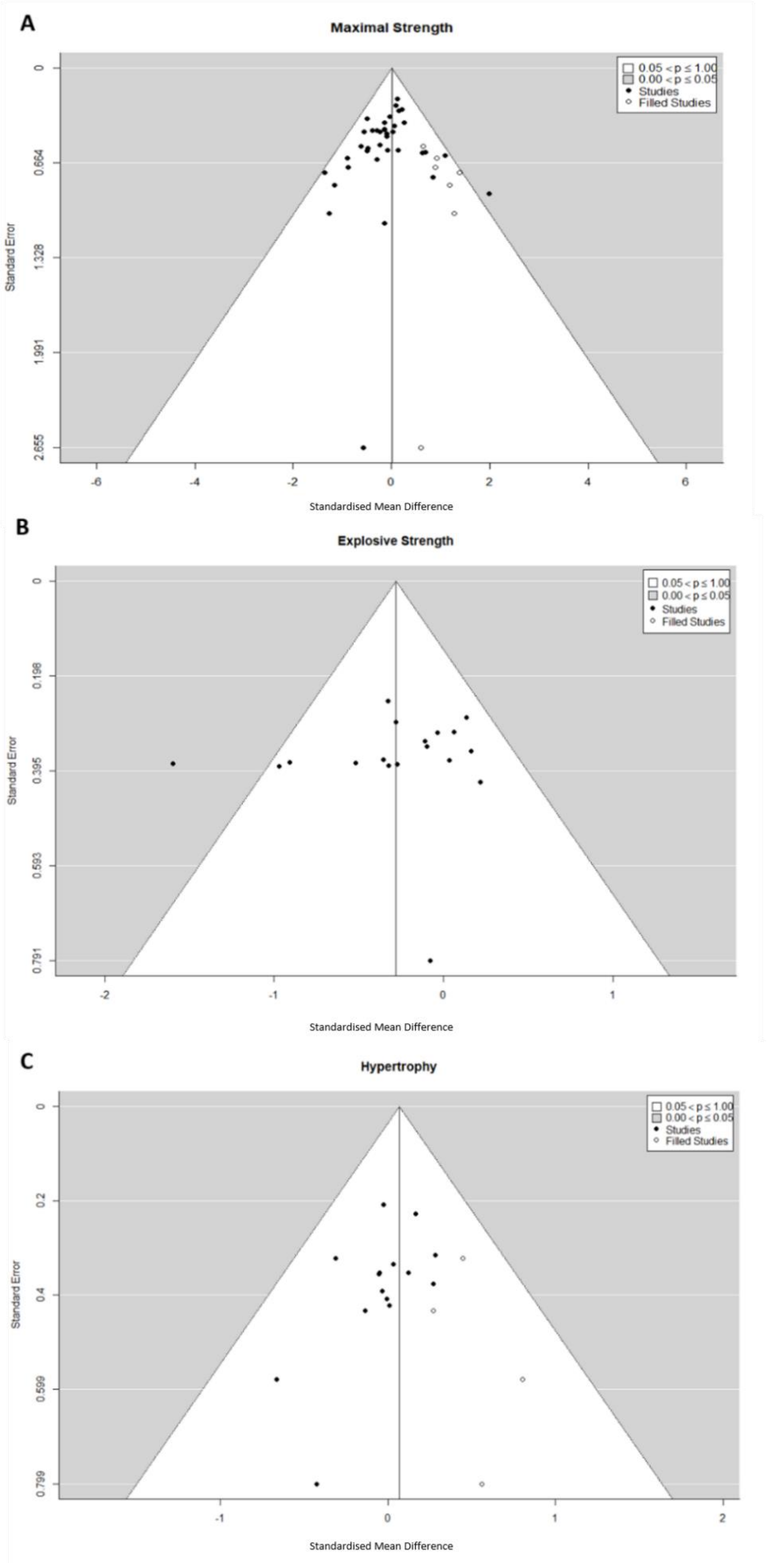

Figure S1. Trim-and-fill funnel plot for maximal strength (**A**), explosive strength (**B**), whole muscle hypertrophy (**C**). Neither the rank correlation nor the regression test indicated any funnel plot asymmetry ($p \geq 0.140$ and $p \geq 0.219$, respectively).

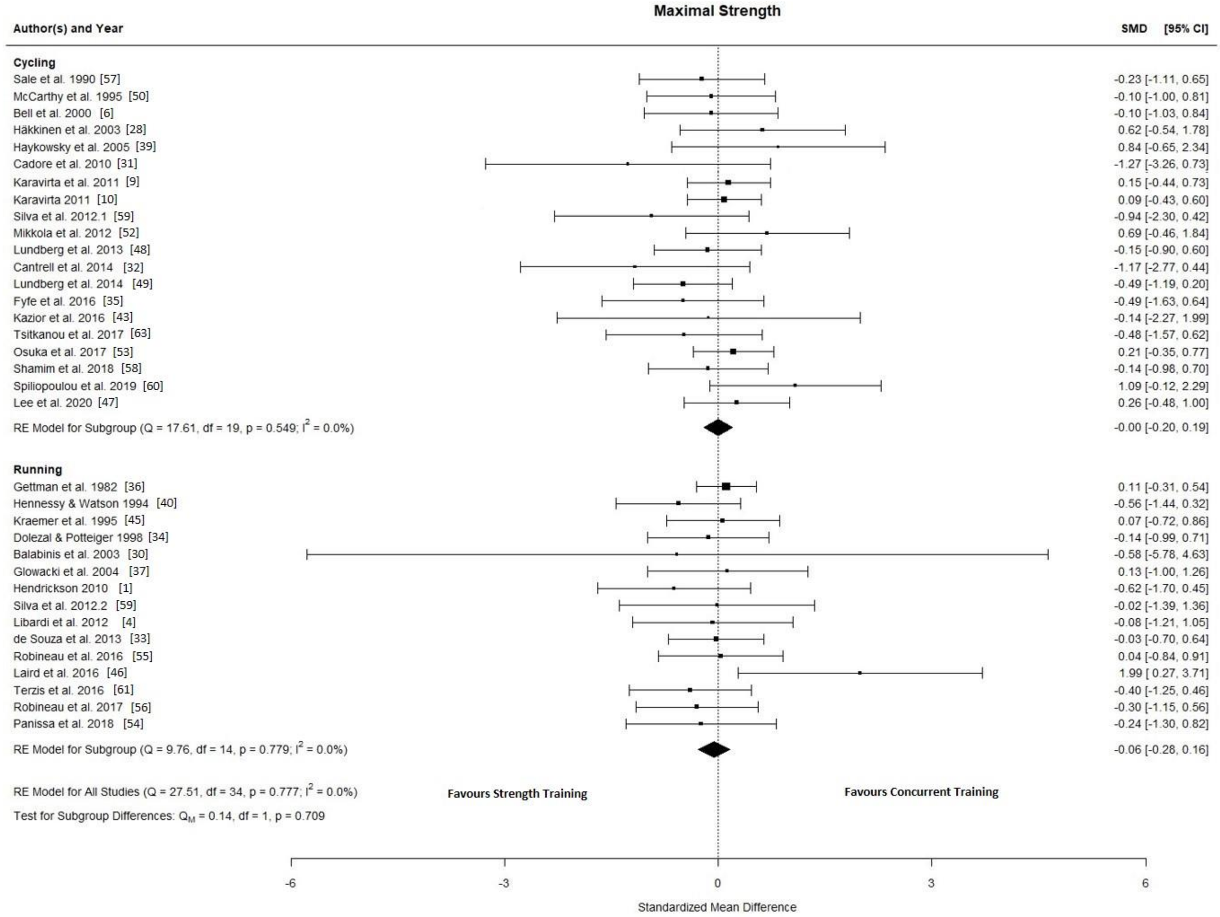

Figure S2. Forest plot of standardised mean differences in maximal strength based on the type of aerobic training. SMD = standardised mean difference; CI = confidence interval.

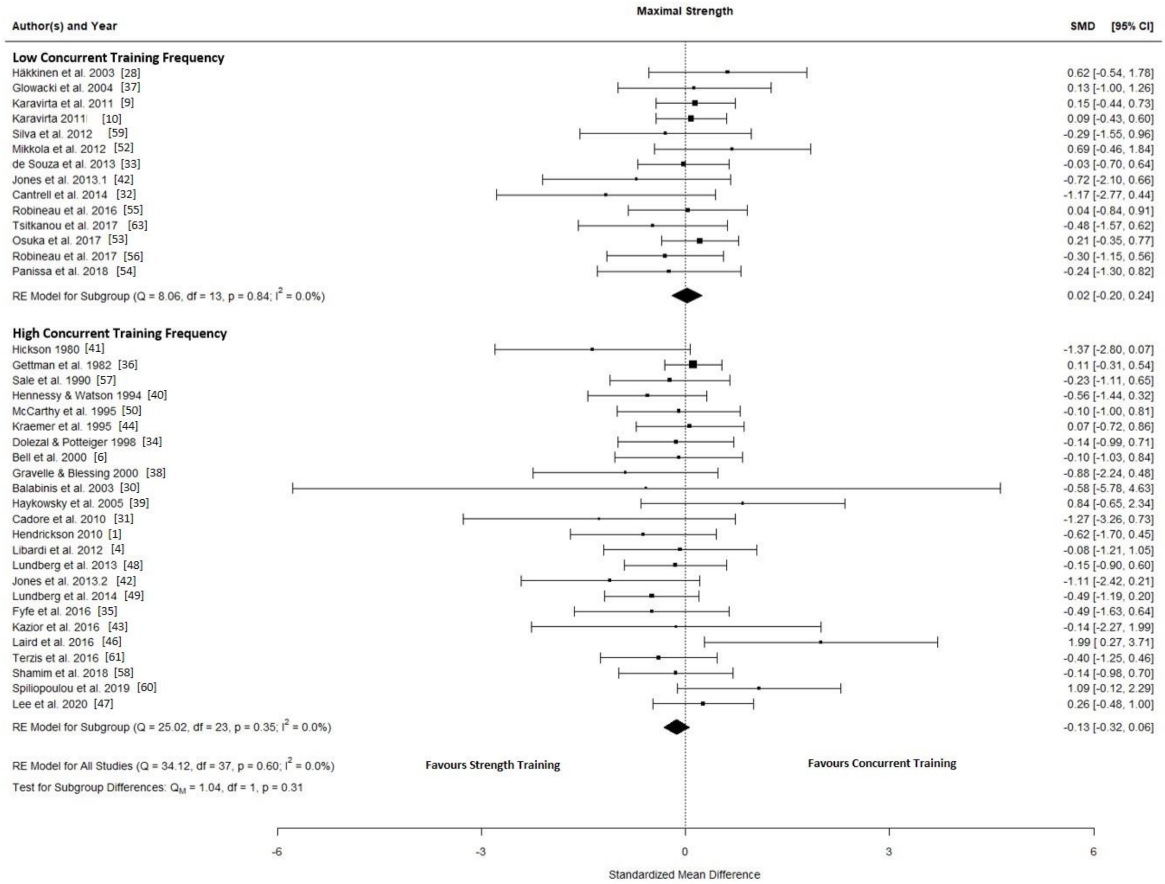

Figure S3. Forest plot of standardised mean differences in maximal strength with low frequency of 4.1 ± 0.3 vs. high frequency of 6.1 ± 1.6 weekly sessions, relating to 2.0 ± 0.3 vs. 3.1 ± 0.6 weekly sessions in the strength training group. SMD = standardised mean difference; CI = confidence interval.

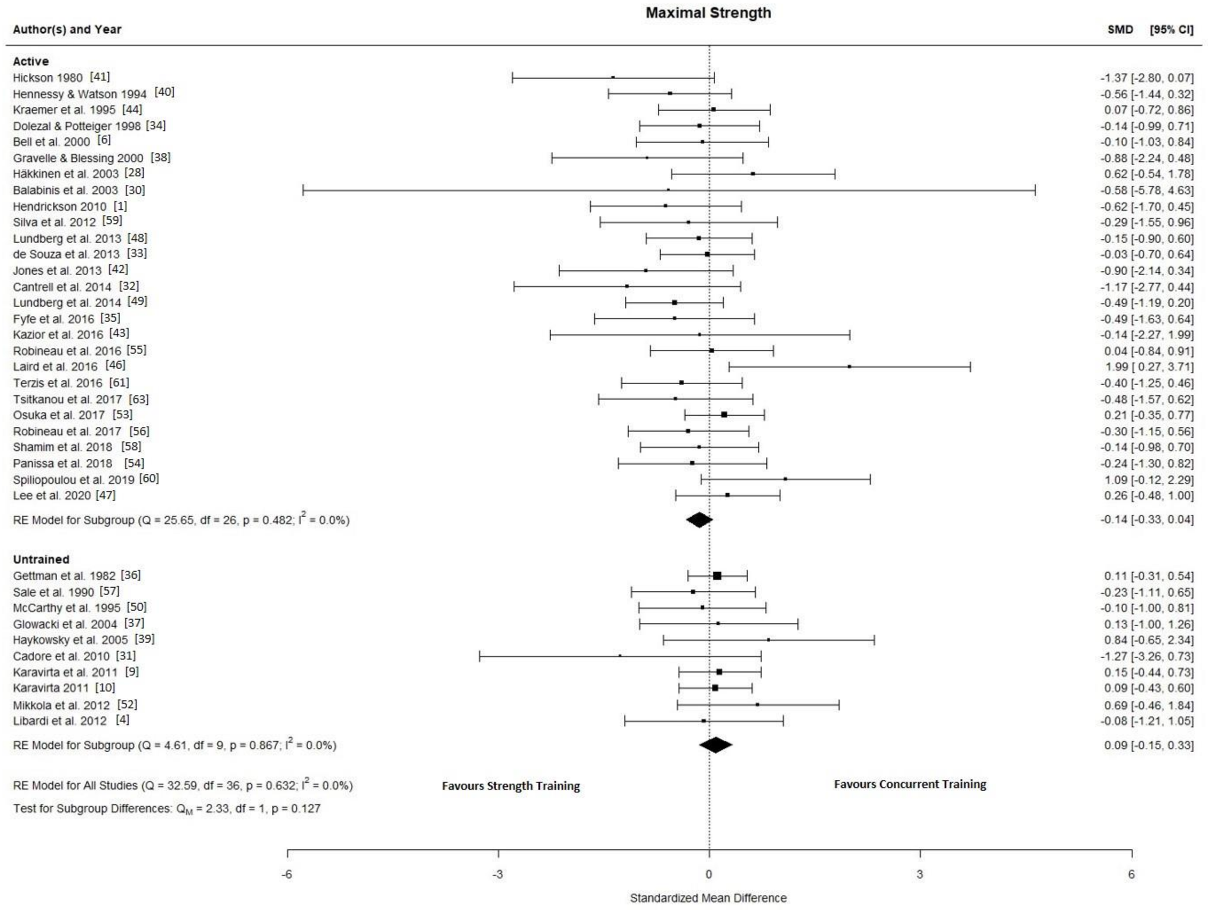

Figure S4. Forest plot of standardised mean differences in maximal strength in untrained vs. active participants. SMD = standardised mean difference; CI = confidence interval.

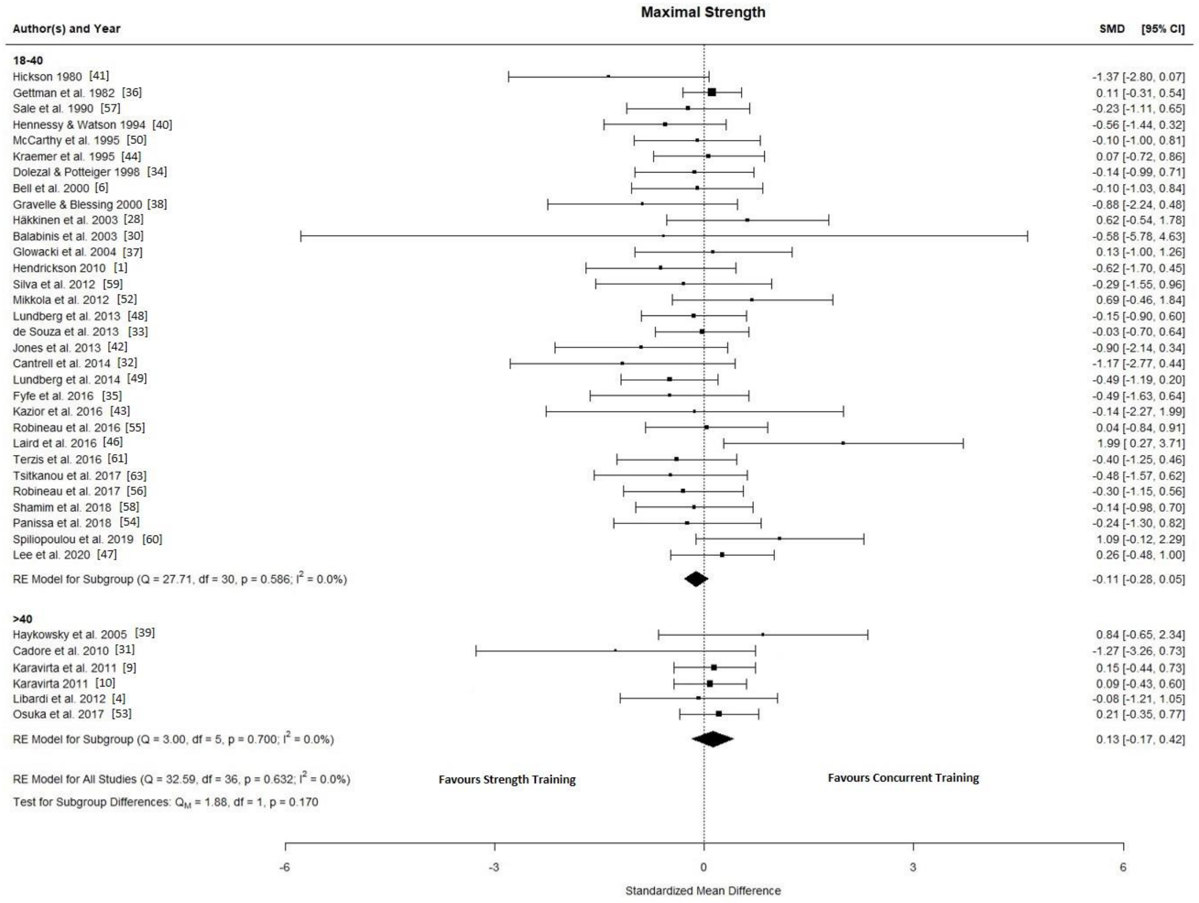

Figure S5. Forest plot of standardised mean differences in maximal strength for participants aged 18-40 vs. >40. SMD = standardised mean difference; CI = confidence interval.

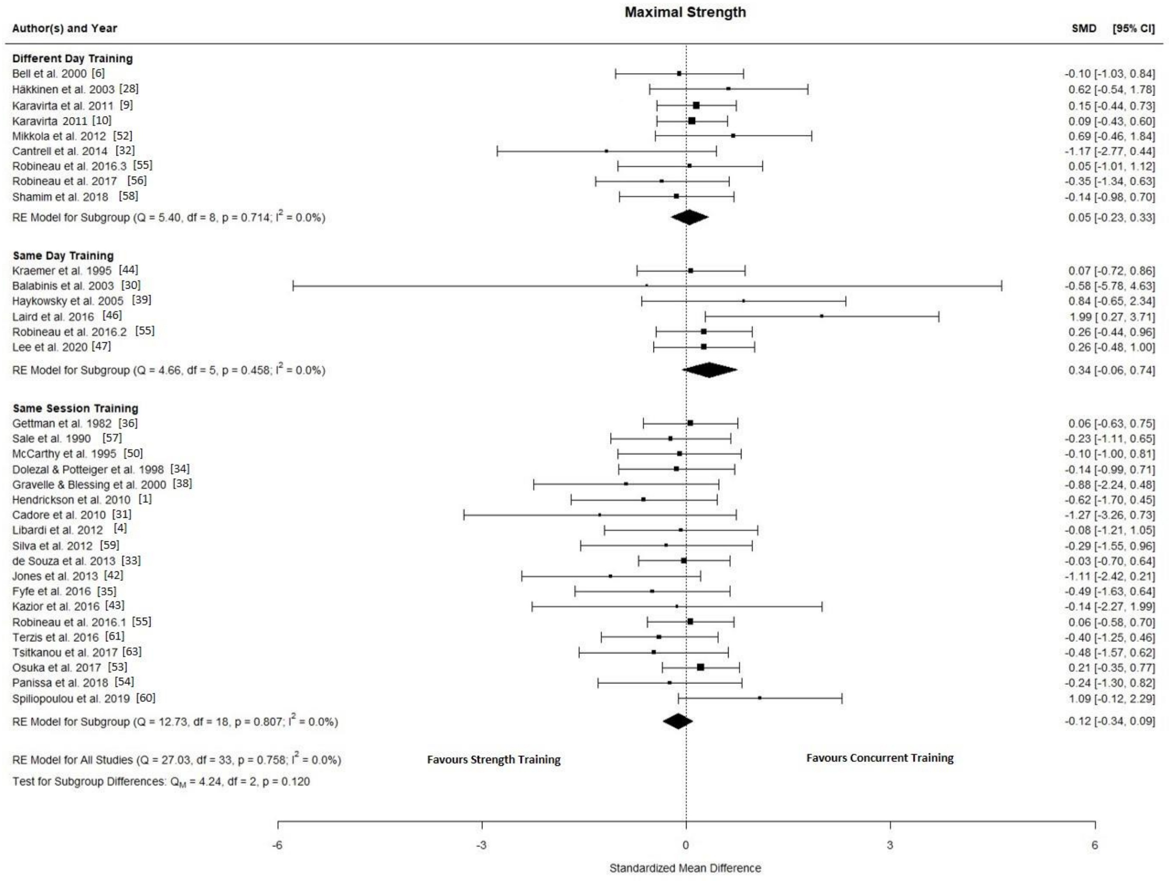

Figure S6. Forest plot of standardised mean differences in maximal strength following concurrent training performed on different days vs. the same day vs. the same session. SMD = standardised mean difference; CI = confidence interval.

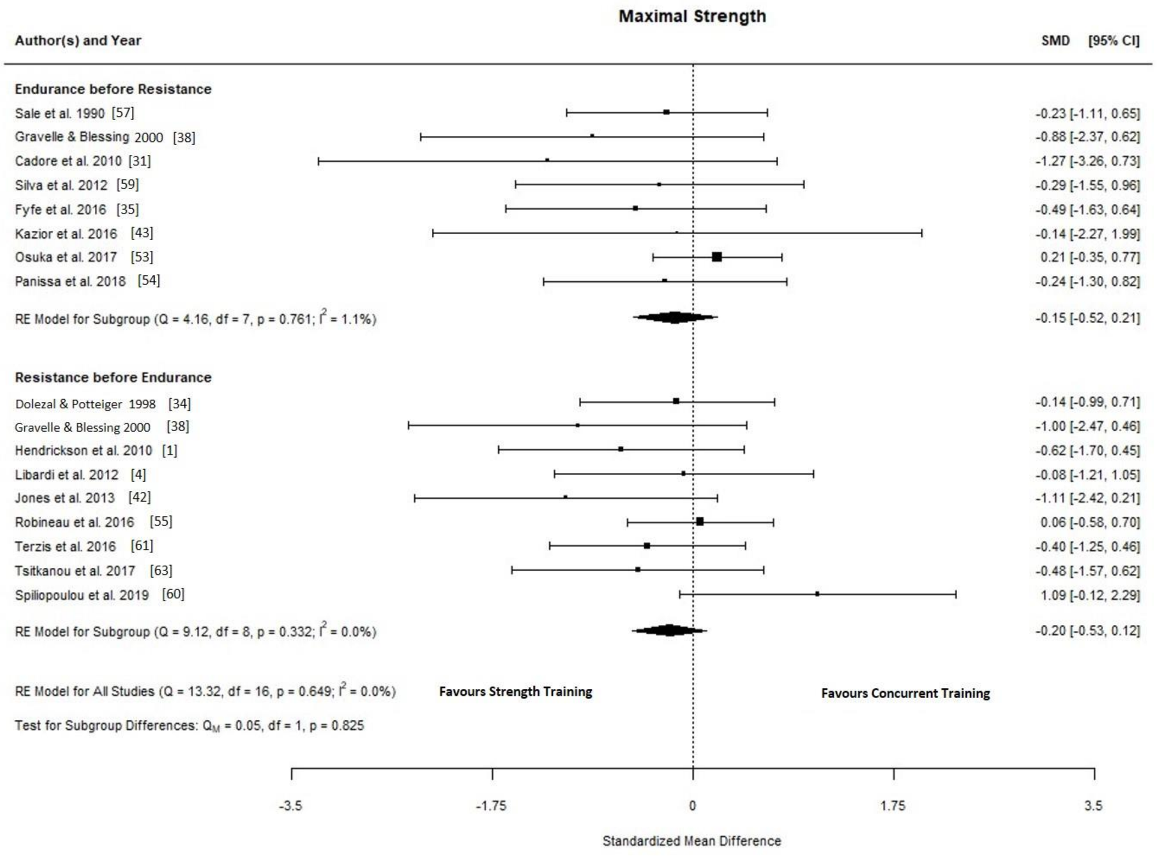

Figure S7. Forest plot of standardised mean differences in maximal strength separated for the training order of same-session training (i.e. aerobic training performed before strength training vs. strength training performed before aerobic training). SMD = standardised mean difference; CI = confidence interval.

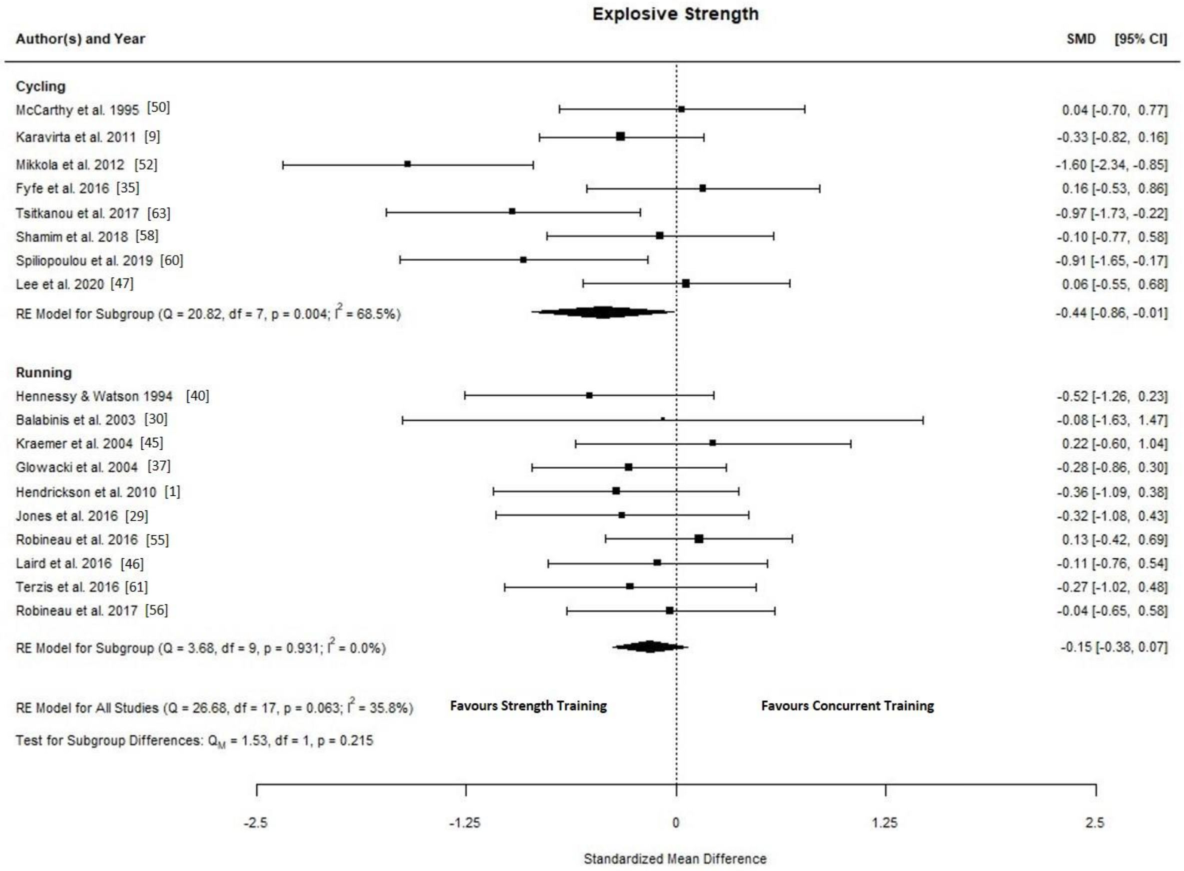

Figure S8. Forest plot of standardised mean differences in explosive strength based on the type of aerobic training. SMD = standardised mean difference; CI = confidence interval.

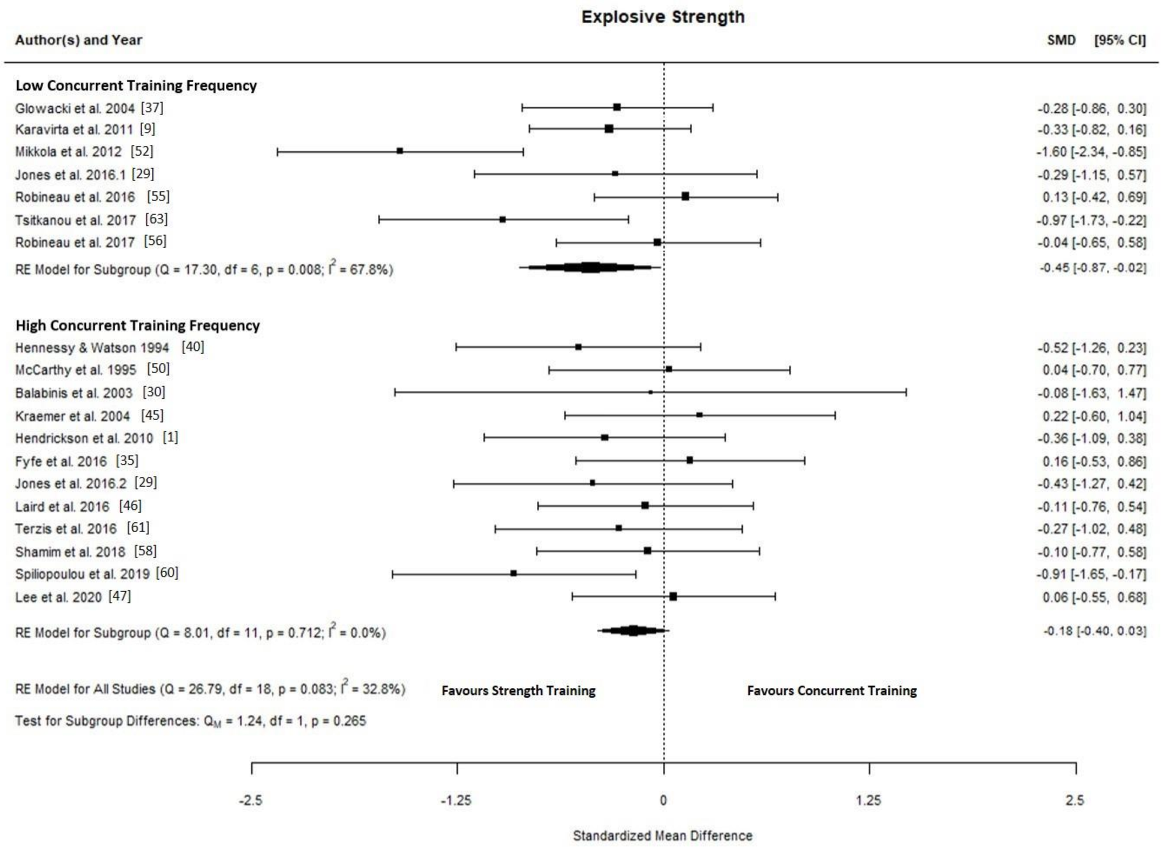

Figure S9. Forest plot of standardised mean differences in explosive strength with low frequency of 4.1 ± 0.3 vs. high frequency of 6.1 ± 1.6 weekly sessions, relating to 2.0 ± 0.3 vs. 3.1 ± 0.6 weekly sessions in the strength training group. SMD = standardised mean difference; CI = confidence interval.

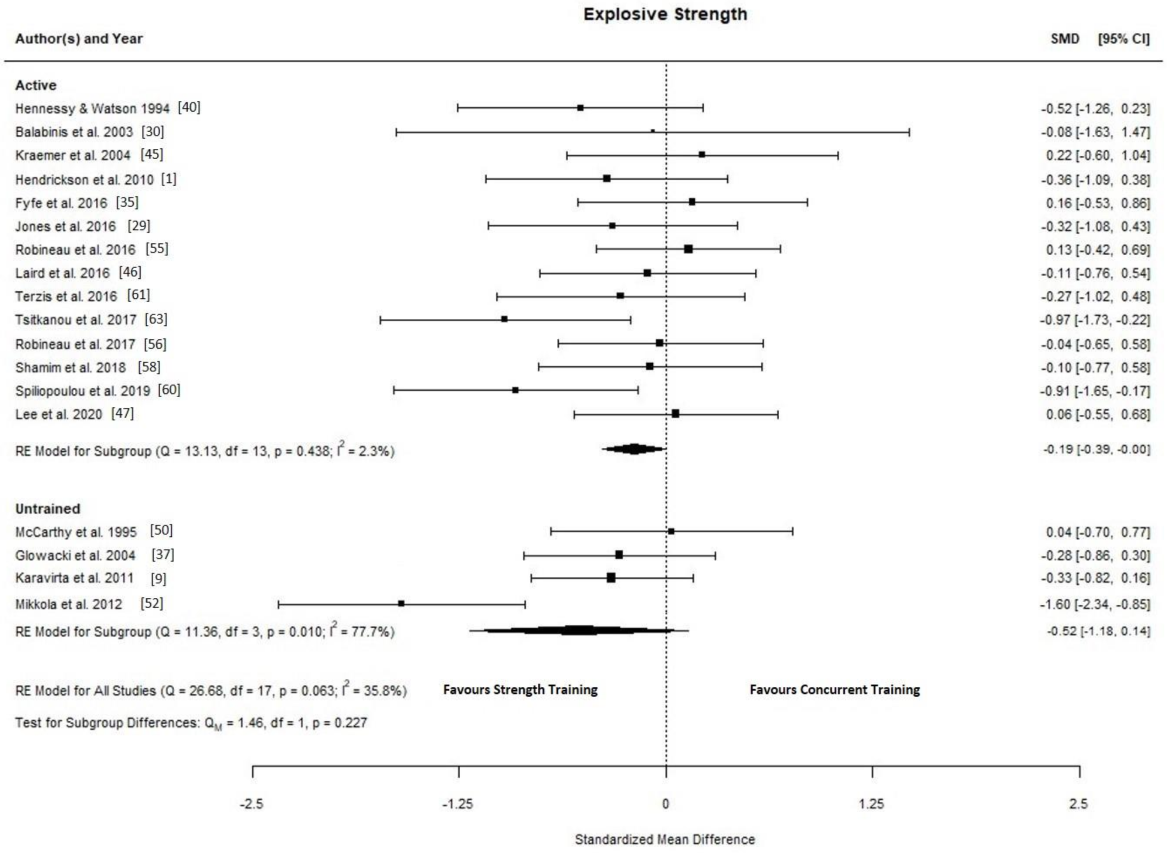

Figure S10. Forest plot of standardised mean differences in explosive strength in untrained vs. active participants. SMD = standardised mean difference; CI = confidence interval.

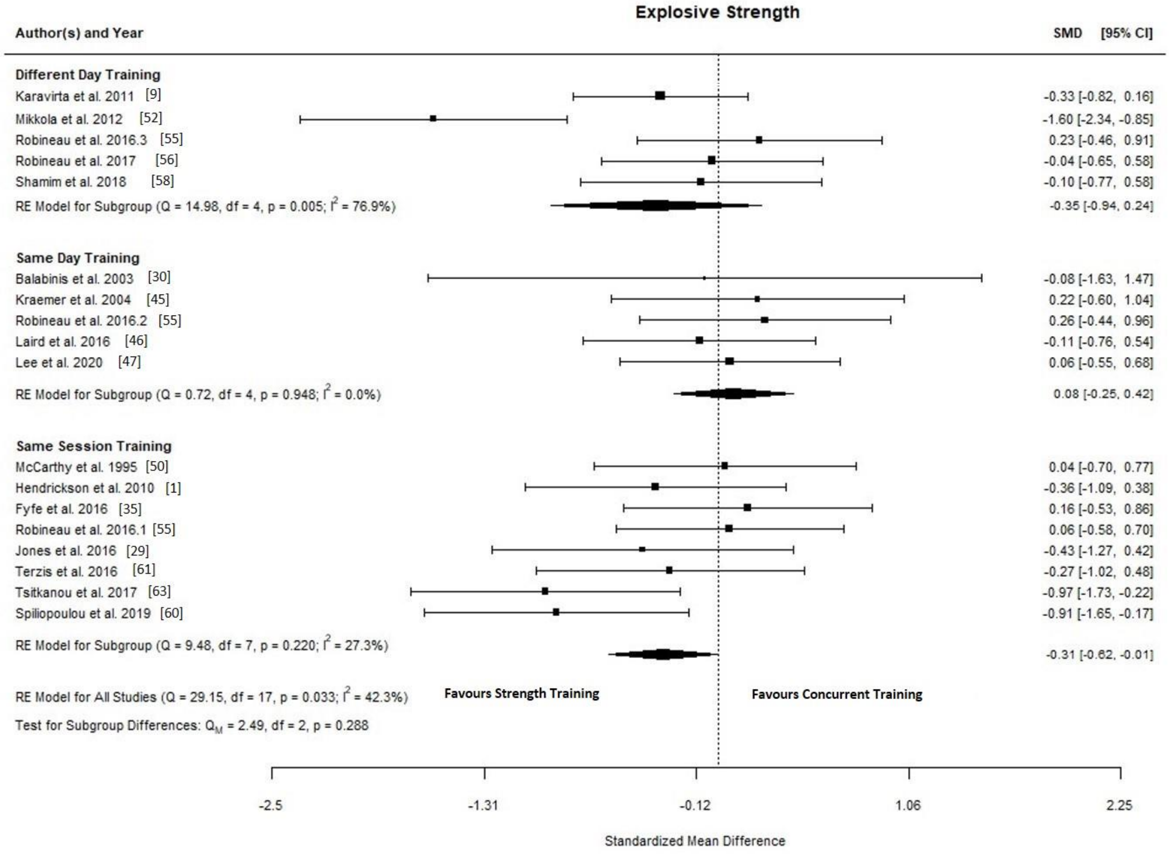

Figure S11. Forest plot of standardised mean differences in explosive strength following different day training vs. same day training vs. same session training. SMD = standardised mean difference; CI = confidence interval.

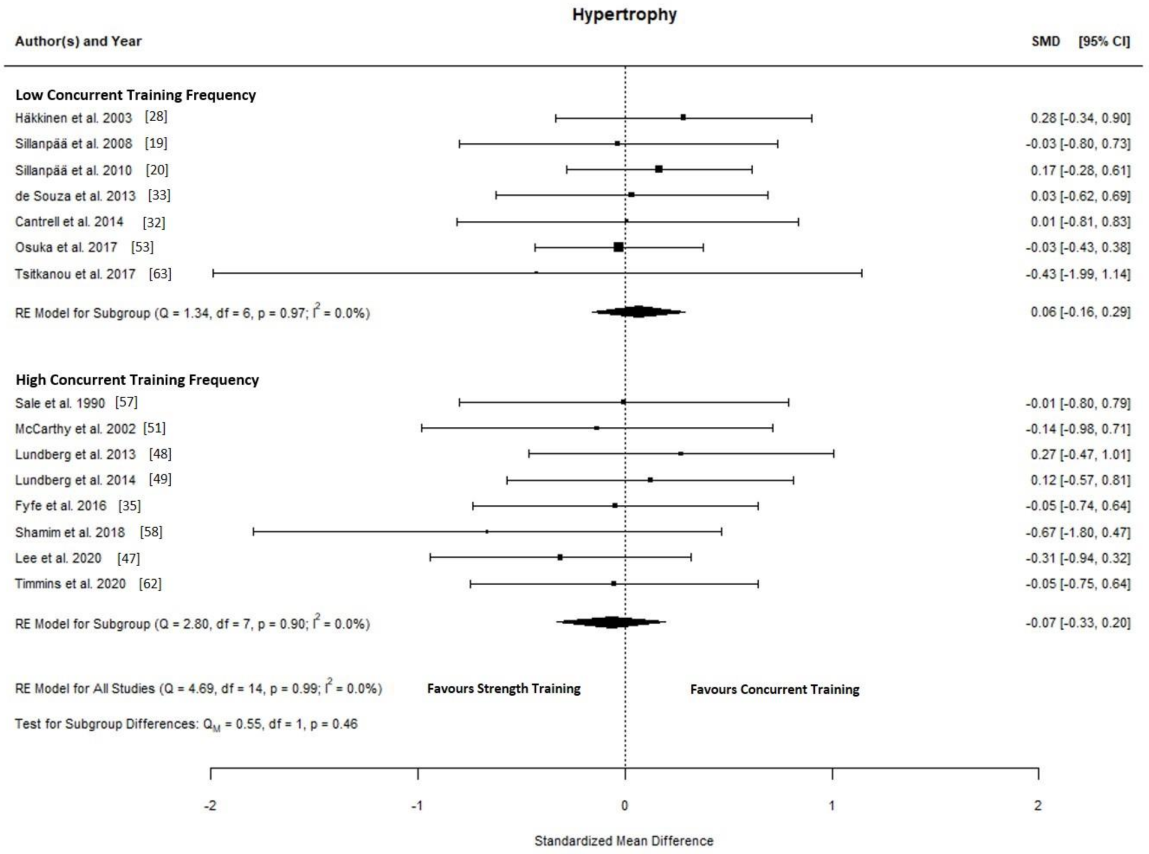

Figure S12. Forest plot of standardised mean differences in muscle hypertrophy with low frequency of 4.1 ± 0.3 vs. high frequency of 6.1 ± 1.6 weekly sessions, relating to 2.0 ± 0.3 vs. 3.1 ± 0.6 weekly sessions in the strength training group. SMD = standardised mean difference; CI = confidence interval.

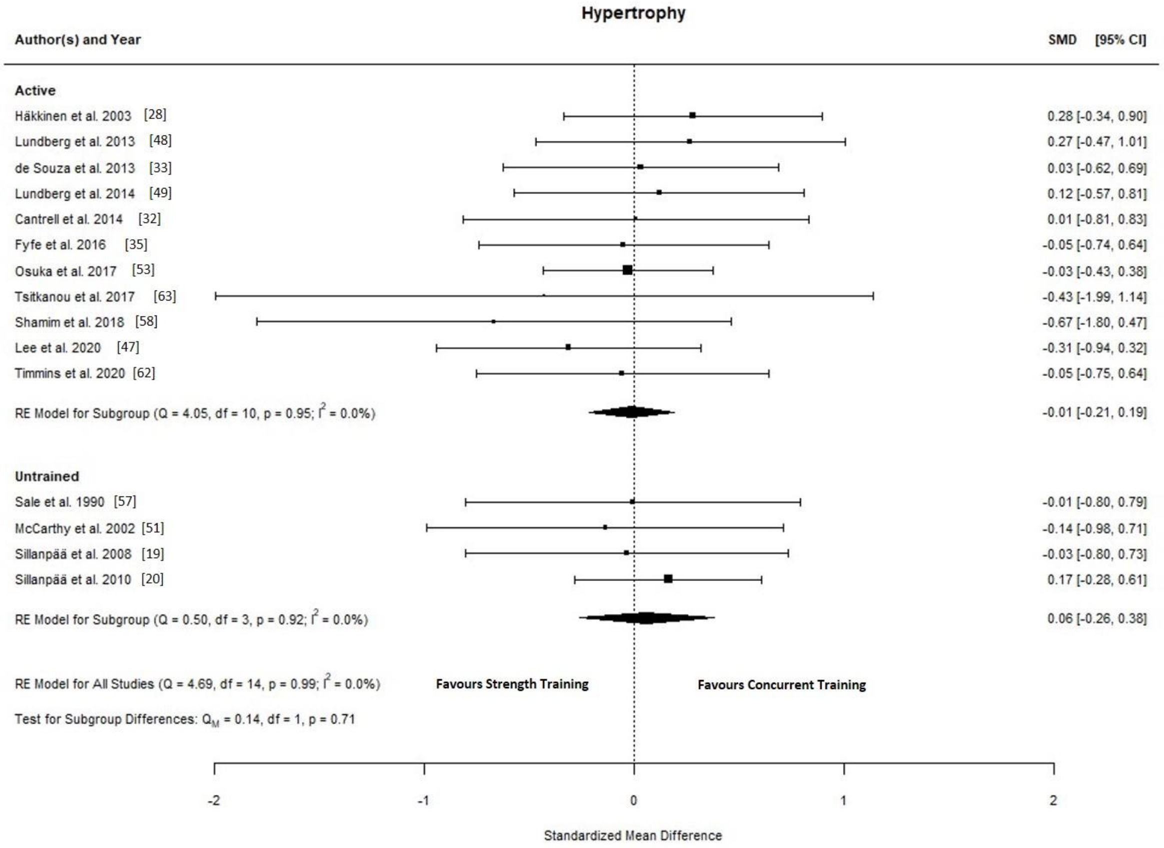

Figure S13. Forest plot of standardised mean differences in muscle hypertrophy in active vs. untrained participants. SMD = standardised mean difference; CI = confidence interval.

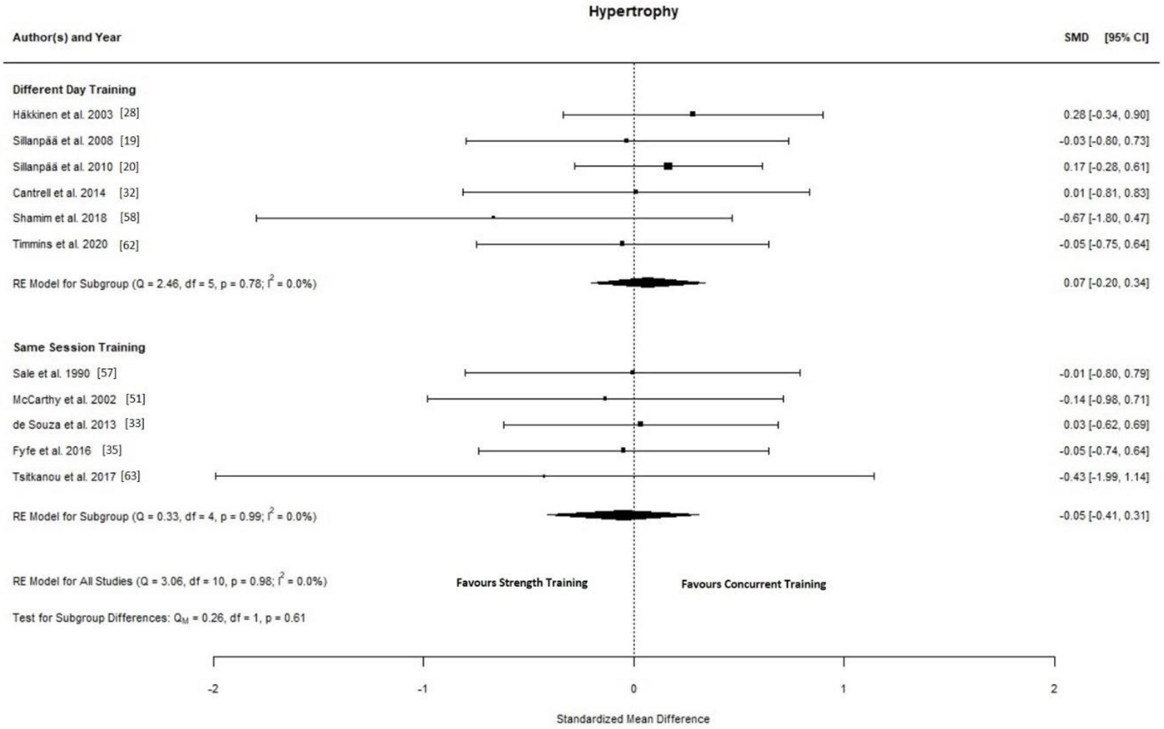

Figure S14. Forest plot of standardised mean differences in muscle hypertrophy following different day training vs. same day training. SMD = standardised mean difference; CI = confidence interval.

References

1. Hendrickson NR, Sharp MA, Alemany JA, Walker LA, Harman EA, Spiering BA, et al. Combined resistance and endurance training improves physical capacity and performance on tactical occupational tasks. Eur J Appl Physiol. 2010;109:1197–208. doi:10.1007/s00421-010-1462-2.

2. Nindl BC, Alemany JA, Tuckow AP, Rarick KR, Staab JS, Kraemer WJ, et al. Circulating bioactive and immunoreactive IGF-I remain stable in women, despite physical fitness improvements after 8 weeks of resistance, aerobic, and combined exercise training. J Appl Physiol (1985). 2010;109:112–20. doi:10.1152/japplphysiol.00025.2010.

3. DuPont WH, Kraemer WJ, Nindl BC, Lee EC, Fragala MS, Hatfield DL, et al. The effects of different exercise training modalities on plasma proenkephalin Peptide F in women. Peptides. 2017;91:26–32. doi:10.1016/j.peptides.2017.02.006.

4. Libardi CA, Souza GV de, Cavaglieri CR, Madruga VA, Chacon-Mikahil MP. Effect of resistance, endurance, and concurrent training on TNF-α, IL-6, and CRP. Med Sci Sports Exerc. 2012;44:50–6. doi:10.1249/MSS.0b013e318229d2e9.

5. Libardi CA, Souza GV, Gáspari AF, Dos Santos CF, Leite ST, Dias R, et al. Effects of concurrent training on interleukin-6, tumour necrosis factor-alpha and C-reactive protein in middle-aged men. J Sports Sci. 2011;29:1573–81. doi: 10.1080/02640414.2011.609896

6. Bell GJ, Syrotuik D, Martin TP, Burnham R, Quinney HA. Effect of concurrent strength and endurance training on skeletal muscle properties and hormone concentrations in humans. Eur J Appl Physiol. 2000;81:418–27. doi:10.1007/s004210050063.

7. Horne L, Bell G, Fisher B, Warren S, Janowska-Wieczorek R. Interaction Between Cortisol and Tumour Necrosis Factor with Concurrent Resistance and Endurance Training. Clinical Journal of Sport Medicine. 1997;7:247–51. doi:10.1097/00042752-199710000-00001.

8. Putman CT, Xu X, Gillies E, MacLean IM, Bell GJ. Effects of strength, endurance and combined training on myosin heavy chain content and fibre-type distribution in humans. Eur J Appl Physiol. 2004;92:376–84. doi:10.1007/s00421-004-1104-7.

9. Karavirta L, Häkkinen A, Sillanpää E, García-López D, Kauhanen A, Haapasaari A, et al. Effects of combined endurance and strength training on muscle strength, power and hypertrophy in 40-67-year-old men. Scandinavian Journal of Medicine and Science in Sports. 2011;21:402–11. doi:10.1111/j.1600-0838.2009.01059.x.

10. Karavirta L. Cardiorespiratory, Neuromuscular and Cardiac Autonomic Adaptations to Combined Endurance and Strength Training in Ageing Men and Women. PhD Thesis, http://urn.fi/URN:ISBN:978-951-39-4216-8.

11. Holviala J, Häkkinen A, Karavirta L, Nyman K, Izquierdo M, Gorostiaga EM, et al. Effects of combined strength and endurance training on treadmill load carrying walking performance in aging men. J Strength Cond Res. 2010;24:1584–95. doi:10.1519/JSC.0b013e3181dba178.

12. Holviala J, Kraemer WJ, Sillanpää E, Karppinen H, Avela J, Kauhanen A, et al. Effects of strength, endurance and combined training on muscle strength, walking speed and dynamic

balance in aging men. Eur J Appl Physiol. 2012;112:1335–47. doi:10.1007/s00421-011-2089-7.

13. Ahtiainen JP, Hulmi JJ, Kraemer WJ, Lehti M, Pakarinen A, Mero AA, et al. Stength, Endurance or Combined Training Elicit Diverse Skeletal Muscle Myosin Heavy Chain Isoform Proportion but Unaltered Androgen Receptor Concentration in Older Men. Int J Sports Med. 2009;30:879–87. doi:10.1055/s-0029-1238290.

14. Sillanpää E, Laaksonen DE, Häkkinen A, Karavirta L, Jensen B, Kraemer WJ, et al. Body composition, fitness, and metabolic health during strength and endurance training and their combination in middle-aged and older women. Eur J Appl Physiol. 2009;106:285–96. doi:10.1007/s00421-009-1013-x.

15. Sillanpää E, Häkkinen A, Punnonen K, Häkkinen K, Laaksonen DE. Effects of strength and endurance training on metabolic risk factors in healthy 40-65-year-old men. Scandinavian Journal of Medicine and Science in Sports. 2009;19:885–95. doi:10.1111/j.1600-0838.2008.00849.x.

16. Sillanpää E, Häkkinen K, Holviala J, Häkkinen A. Combined strength and endurance training improves health-related quality of life in healthy middle-aged and older adults. Int J Sports Med. 2012;33:981–6. doi:10.1055/s-0032-1311589.

17. Karavirta L, Tulppo MP, Laaksonen DE, Nyman K, Laukkanen RT, Kinnunen H, et al. Heart rate dynamics after combined endurance and strength training in older men. Med Sci Sports Exerc. 2009;41:1436–43. doi:10.1249/MSS.0b013e3181994a91.

18. Karavirta L, Costa MD, Goldberger AL, Tulppo MP, Laaksonen DE, Nyman K, et al. Heart Rate Dynamics after Combined Strength and Endurance Training in Middle-Aged Women: Heterogeneity of Responses. PLoS One 2013;8: e72664 doi:10.1371/journal.pone.0072664.

19. Sillanpää E, Häkkinen A, Nyman K, Mattila M, Cheng S, Karavirta L, et al. Body composition and fitness during strength and/or endurance training in older men. Med Sci Sports Exerc. 2008;40:950–8. doi:10.1249/MSS.0b013e318165c854.

20. Sillanpää E, Häkkinen A, Laaksonen DE, Karavirta L, Kraemer WJ, Häkkinen K. Serum basal hormone concentrations, nutrition and physical fitness during strength and/or endurance training in 3964-year-old women. Int J Sports Med. 2010;31:110–7. doi:10.1055/s-0029-1242811.

21. Chtara M, Chaouachi A, Levin GT, Chaouachi M, Chamari K, Amri M, Laursen PB. Effect of concurrent endurance and circuit resistance training sequence on muscular strength and power development. J Strength Cond Res. 2008;22:1037–45. doi:10.1519/JSC.0b013e31816a4419.

22. Coleman EA, Buchner DM, Cress ME, Chan BK, Lateur BJ de. The relationship of joint symptoms with exercise performance in older adults. J Am Geriatr Soc. 1996;44:14–21. doi:10.1111/j.1532-5415.1996.tb05632.x.

23. Dudley GA, Djamil R. Incompatibility of endurance- and strength-training modes of exercise. J Appl Physiol (1985). 1985;59:1446–51. doi:10.1152/jappl.1985.59.5.1446.

24. Gergley JC. Comparison of two lower-body modes of endurance training on lower-body strength development while concurrently training. J Strength Cond Res. 2009;23:979–87. doi:10.1519/JSC.0b013e3181a0629d.

25. Lambert BS, Shimkus KL, Fluckey JD, Riechman SE, Greene NP, Cardin JM, Crouse SF. Anabolic responses to acute and chronic resistance exercise are enhanced when combined with aquatic treadmill exercise. American journal of physiology. Endocrinology and metabolism. 2015;308:E192--E200. doi:10.1152/ajpendo.00689.2013.

26. Leveritt M, Abernethy PJ, Barry B, Logan PA. Concurrent strength and endurance training: The influence of dependent variable selection. J Strength Cond Res 2003;17:503-508. doi:10.1519/1533-4287(2003)017<0503:CSAETT>2.0.CO;2.

27. Peres Campos AL, Del Ponte, Lourenço dos Santos, Schüler Cavalli A, da Rosa Afonso M, Gomes Schild JF, Fossati Reichert F. Effects of concurrent training on health aspects of elderly women. Revista Brasileira de Cineantropometria e Desempenho Humano. 2013;15:437–47. doi:10.5007/1980-0037.2013v15n4p437.

28. Häkkinen K, Alen M, Kraemer WJ, Gorostiaga E, Izquierdo M, Rusko H, et al. Neuromuscular adaptations during concurrent strength and endurance training versus strength training. Eur J Appl Physiol. 2003;89:42–52. doi:10.1007/s00421-002-0751-9.

29. Jones TW, Howatson G, Russell M, French DN. Performance and Endocrine Responses to Differing Ratios of Concurrent Strength and Endurance Training. J Strength Cond Res. 2016;30:693–702. doi:10.1519/JSC.0000000000001135.

30. Balabinis CP, Psarakis CH, Moukas M, Vassiliou MP, Behrakis PK. Early phase changes by concurrent endurance and strength training. J Strength Cond Res. 2003;17:393–401. doi:10.1519/1533-4287(2003)017<0393:epcbce>2.0.co;2.

31. Cadore EL, Pinto RS, Lhullier FLR, Correa CS, Alberton CL, Pinto SS, et al. Physiological effects of concurrent training in elderly men. Int J Sports Med. 2010;31:689–97. doi:10.1055/s-0030-1261895.

32. Cantrell GS, Schilling BK, Paquette MR, Murlasits Z. Maximal strength, power, and aerobic endurance adaptations to concurrent strength and sprint interval training. Eur J Appl Physiol. 2014;114:763–71. doi:10.1007/s00421-013-2811-8.

33. Souza EO de, Tricoli V, Roschel H, Brum PC, Bacurau AVN, Ferreira JCB, et al. Molecular adaptations to concurrent training. Int J Sports Med. 2013;34:207–13. doi:10.1055/s-0032-1312627.

34. Dolezal BA, Potteiger JA. Concurrent resistance and endurance training influence basal metabolic rate in nondieting individuals. J Appl Physiol (1985). 1998;85:695–700. doi:10.1152/jappl.1998.85.2.695.

35. Fyfe JJ, Bartlett JD, Hanson ED, Stepto NK, Bishop DJ. Endurance training intensity does not mediate interference to maximal lower-body strength gain during short-term concurrent training. Frontiers in Physiology 2016. doi:10.3389/fphys.2016.00487.

36. Gettman LR, Ward P, Hagan RD. A comparison of combined running and weight training with circuit weight training. Med Sci Sports Exerc. 1982;14:229–34.

37. Glowacki SP, Martin SE, Maurer A, Baek W, Green JS, Crouse SF. Effects of resistance, endurance, and concurrent exercise on training outcomes in men. Med Sci Sports Exerc. 2004;36:2119–27. doi:10.1249/01.MSS.0000147629.74832.52.

38. Gravelle BL, Blessing DL. Physiological Adaptation in Women Concurrently Training for Strength and Endurance. J Strength Cond Res. 2000;14:5–13. doi:10.1519/00124278-200002000-00002.

39. Haykowsky M, McGavock J, Muhll IV, Koller M, Mandic S, Welsh R, et al. Effect of Exercise Training on Peak Aerobic Power, Left Ventricular Morphology, and Muscle Strength in Healthy Older Women. Journals of Gerontology Series A: Biological Sciences & Medical Sciences. 2005;60:307–11. doi:10.1093/gerona/60.3.307.

40. Hennessy LC, Watson AWS. The Interference Effects of Training for Strength and Endurance Simultaneously. The Journal of Strength and Conditioning Research. 1994;8:12. doi:10.1519/1533-4287(1994)008<0012:TIEOTF>2.3.CO;2.

41. Hickson RC. Interference of strength development by simultaneously training for strength and endurance. Eur J Appl Physiol Occup Physiol. 1980;45:255–63. doi:10.1007/BF00421333.

42. Jones TW, Howatson G, Russell M, French DN. Performance and neuromuscular adaptations following differing ratios of concurrent strength and endurance training. J Strength Cond Res. 2013;27:3342–51. doi:10.1519/JSC.0b013e3181b2cf39.

43. Kazior Z, Willis SJ, Moberg M, Apró W, Calbet JAL, Holmberg HC, Blomstrand E. Endurance exercise enhances the effect of strength training on muscle fiber size and protein expression of akt and mTOR. PLoS One. 2016; 17;11:e0149082, doi:10.1371/journal.pone.0149082.

44. Kraemer WJ, Patton JF, Gordon SE, Harman EA, Deschenes MR, Reynolds K, et al. Compatibility of high-intensity strength and endurance training on hormonal and skeletal muscle adaptations. J Appl Physiol (1985). 1995;78:976–89. doi:10.1152/jappl.1995.78.3.976.

45. Kraemer WJ, Vescovi JD, Volek JS, Nindl BC, Newton RU, Patton JF, et al. Effects of concurrent resistance and aerobic training on load-bearing performance and the Army physical fitness test. Military Medicine. 2004;169:994–9. doi:10.7205/milmed.169.12.994.

46. Laird RH, Elmer DJ, Barberio MD, Salom LP, Lee KA, Pascoe DD. Evaluation of Performance Improvements After Either Resistance Training or Sprint Interval-Based Concurrent Training. J Strength Cond Res. 2016;30:3057–65. doi:10.1519/JSC.0000000000001412.

47. Lee MJ-C, Ballantyne JK, Chagolla J, Hopkins WG, Fyfe JJ, Phillips SM, et al. Order of same-day concurrent training influences some indices of power development, but not strength, lean mass, or aerobic fitness in healthy, moderately-active men after 9 weeks of training. PLoS One. 2020;15:e0233134. doi:10.1371/journal.pone.0233134.

48. Lundberg TR, Fernandez-Gonzalo R, Gustafsson T, Tesch PA. Aerobic exercise does not compromise muscle hypertrophy response to short-term resistance training. J Appl Physiol (1985). 2013;114:81–9. doi:10.1152/japplphysiol.01013.2012.

49. Lundberg TR, Fernandez-Gonzalo R, Tesch PA. Exercise-induced AMPK activation does not interfere with muscle hypertrophy in response to resistance training in men. J Appl Physiol (1985). 2014;116:611–20. doi:10.1152/japplphysiol.01082.2013.

50. McCarthy JP, Agre JC, Graf BK, Pozniak MA, Vailas AC. Compatibility of adaptive responses with combining strength and endurance training. Med Sci Sports Exerc. 1995;27:429–36.

51. McCarthy JP, Pozniak MA, Agre JC. Neuromuscular adaptations to concurrent strength and endurance training. Med Sci Sports Exerc. 2002;34:511–9. doi:10.1097/00005768-200203000-00019.

52. Mikkola J, Rusko H, Izquierdo M, Gorostiaga EM, Häkkinen K. Neuromuscular and cardiovascular adaptations during concurrent strength and endurance training in untrained men. Int J Sports Med. 2012;33:702–10. doi:10.1055/s-0031-1295475.

53. Osuka Y, Fujita S, Kitano N, Kosaki K, Seol J, Sawano Y, et al. Effects of Aerobic and Resistance Training Combined with Fortified Milk on Muscle Mass, Muscle Strength, and Physical Performance in Older Adults: A Randomized Controlled Trial. The journal of nutrition, health & aging. 2017;21:1349–57. doi:10.1007/s12603-016-0864-1.

54. Panissa VLG, Fukuda DH, Oliveira FP de, Parmezzani SS, Campos EZ, Rossi FE, et al. Maximum Strength Development and Volume-Load during Concurrent High Intensity Intermittent Training Plus Strength or Strength-Only Training. Journal of sports science & medicine. 2018;17:623–32.

55. Robineau J, Babault N, Piscione J, Lacome M, Bigard A. Specific Training Effects of Concurrent Aerobic and Strength Exercises Depend on Recovery Duration. J Strength Cond Res. 2016;30:672–83. doi:10.1519/JSC.0000000000000798.

56. Robineau J, Lacome M, Piscione J, Bigard X, Babault N. Concurrent Training in Rugby Sevens: Effects of High-Intensity Interval Exercises. International Journal of Sports Physiology & Performance. 2017;12:336–44. doi:10.1123/ijspp.2015-0370.

57. Sale DG, MacDougall JD, Jacobs I, Garner S. Interaction between concurrent strength and endurance training. Journal of Applied Physiology. 1990;68:260–70. doi: 10.1152/jappl.1990.68.1.260

58. Shamim B, Devlin BL, Timmins RG, Tofari P, Lee Dow C, Coffey VG, et al. Adaptations to Concurrent Training in Combination with High Protein Availability: A Comparative Trial in Healthy, Recreationally Active Men. Sports Med. 2018;48:2869–83. doi:10.1007/s40279-018-0999-9.

59. Silva RF, Cadore EL, Kothe G, Guedes M, Alberton CL, Pinto SS, et al. Concurrent training with different aerobic exercises. Int J Sports Med. 2012;33:627–34. doi:10.1055/s-0031-1299698.

60. Spiliopoulou P, Zaras N, Methenitis S, Papadimas G, Papadopoulos C, Bogdanis GC, Terzis G. Effect of Concurrent Power Training and High-Intensity Interval Cycling on Muscle Morphology and Performance. J Strength Cond Res 2019, doi:10.1519/JSC.0000000000003172.

61. Terzis G, Spengos K, Methenitis S, Aagaard P, Karandreas N, Bogdanis G. Early phase interference between low-intensity running and power training in moderately trained females. Eur J Appl Physiol. 2016;116:1063–73. doi:10.1007/s00421-016-3369-z.

62. Timmins RG, Shamim B, Tofari PJ, Hickey JT, Camera DM. Differences in Lower Limb Strength and Structure After 12 Weeks of Resistance, Endurance, and Concurrent Training. International journal of sports physiology and performance. 2020:1–8. doi:10.1123/ijspp.2019-0788.

63. Tsitkanou S, Spengos K, Stasinaki A-N, Zaras N, Bogdanis G, Papadimas G, Terzis G. Effects of high-intensity interval cycling performed after resistance training on muscle strength and hypertrophy. Scand J Med Sci Sports. 2017;27:1317–27. doi:10.1111/sms.12751.
